# Fueled by methane: deep-sea sponges from asphalt seeps gain their nutrition from methane-oxidizing symbionts

**DOI:** 10.1038/s41396-019-0346-7

**Published:** 2019-01-15

**Authors:** Maxim Rubin-Blum, Chakkiath Paul Antony, Lizbeth Sayavedra, Clara Martínez-Pérez, Daniel Birgel, Jörn Peckmann, Yu-Chen Wu, Paco Cardenas, Ian MacDonald, Yann Marcon, Heiko Sahling, Ute Hentschel, Nicole Dubilier

**Affiliations:** 1 Max Planck Institute for Marine Microbiology, Celsiusstrasse 1, 28359 Bremen, Germany; 2 Israel Limnology and Oceanography Research, Tel Shikmona, 3108000 Haifa, Israel; 3 Quadram Institute Bioscience, Norwich Research Park, Norwich, UK; 4 Institute for Geology, Center for Earth System Research and Sustainability, University of Hamburg, 20146 Hamburg, Germany; 5 GEOMAR Helmholtz Centre for Ocean Research, RD3 Marine Microbiology and Christian-Albrechts University of Kiel, Düsternbrooker Weg 20, D-24105 Kiel, Germany; 6 Department of Medicinal Chemistry, Pharmacognosy, BioMedical Centre, Uppsala University, Husargatan 3, 751 23 Uppsala, Sweden; 7 Florida State University, POB 3064326, Tallahassee, FL 32306 USA; 8 Wegener Institute Helmholtz Centre for Polar and Marine Research, HGF-MPG Group for Deep Sea Ecology and Technology, Am Handelshafen 12, 27570 Bremerhaven, Germany; 9 MARUM, Center for Marine Environmental Sciences, University of Bremen, 28359 Bremen, Germany

**Keywords:** Microbial ecology, Bacterial genetics, Symbiosis

## Abstract

Sponges host a remarkable diversity of microbial symbionts, however, the benefit their microbes provide is rarely understood. Here, we describe two new sponge species from deep-sea asphalt seeps and show that they live in a nutritional symbiosis with methane-oxidizing (MOX) bacteria. Metagenomics and imaging analyses revealed unusually high amounts of MOX symbionts in hosts from a group previously assumed to have low microbial abundances. These symbionts belonged to the Marine Methylotrophic Group 2 clade. They are host-specific and likely vertically transmitted, based on their presence in sponge embryos and streamlined genomes, which lacked genes typical of related free-living MOX. Moreover, genes known to play a role in host–symbiont interactions, such as those that encode eukaryote-like proteins, were abundant and expressed. Methane assimilation by the symbionts was one of the most highly expressed metabolic pathways in the sponges. Molecular and stable carbon isotope patterns of lipids confirmed that methane-derived carbon was incorporated into the hosts. Our results revealed that two species of sponges, although distantly related, independently established highly specific, nutritional symbioses with two closely related methanotrophs. This convergence in symbiont acquisition underscores the strong selective advantage for these sponges in harboring MOX bacteria in the food-limited deep sea.

## Introduction

Symbioses with microorganisms have played a central role in shaping the ecology and evolution of marine animals [1]. Sponges are one of the oldest animal phyla and may have lived in symbiosis with microbial partners for hundreds of million years [2–4]. Most sponge species belong to one of two general categories: High microbial abundance (HMA) sponges such as those from the orders Agelasida and Verongida harbor dense microbial consortia with high phylogenetic diversity, while low microbial abundance (LMA) sponges such as those from the order Poecilosclerida have several orders of magnitude fewer symbionts with low phylogenetic diversity [5]. In both HMA and LMA sponges, the microbial symbionts are hypothesized to increase their host’s fitness, for example by recycling nutrients and producing secondary metabolites that can deter predators [4, 6–8]. To date, the functional diversity of microorganisms associated with sponges has been studied primarily in hosts from shallow-water habitats, using metagenomics [9–15], proteomics [16], and transcriptomics [17–21]. However, the remarkable diversity of the microbiota in most shallow-water sponges [3] has made it highly challenging to understand their functional and ecological role. Even less is known about the metabolism, ecology, and evolutionary history of the microorganisms that live in symbiosis with deep-sea sponges.

In the deep sea, light is insufficient to sustain photosynthetic primary production, the input of particulate organic carbon from the surface is low, and nutrition is often limited [22]. While shallow-water sponges primarily gain their nutrition by filter-feeding on planktonic microorganisms and organic matter [4], some sponges have adapted to the deep sea, where low amounts of particulate organic carbon make filter-feeding energetically unfavorable, by becoming carnivores [23–25]. Sponges from cold seeps and hydrothermal vents in the deep sea may have evolved an alternative nutritional strategy. In these environments, abundant chemosynthetic primary production is fueled by reduced energy sources. Analyses of microbial communities based on metagenomics and 16S rRNA gene amplicon sequencing, as well as carbon stable isotope values, indicated that sponges from seeps are associated with chemosynthetic and hydrocarbon-degrading bacteria [25–30]. Thus, in addition to filter-feeding or carnivory, these sponges could gain a considerable proportion of their nutrition from chemosynthetic symbionts.

In contrast to the well-studied chemosynthetic symbioses of more highly evolved invertebrate groups such as bivalves and annelids [31], little is known about these associations in sponges. To gain a better understanding of chemosynthetic symbioses in deep-sea sponges, we used metagenomics, metatranscriptomics, fluorescence, and electron microscopy, as well as lipid (fatty acids and sterols) and stable isotope analyses to study two sponge species from hydrocarbon seeps at Campeche Knolls at 2900–3100 m depth in the southern Gulf of Mexico. These sites are characterized by prolific asphalt flows, oil seepage, gas hydrates, and gas venting [32]. Campeche sponges were previously shown to host hydrocarbon-degrading *Cycloclasticus* bacteria, but these make up only about 5% of their bacterial community [33]. The majority of the sponge microbiota (50–70%) was dominated by methane-oxidizing (MOX) bacteria (the abbreviation MOX is also used for methane oxidizer(s) in the following), [33]. In this study, we provide in-depth insights into the symbiosis between sponges from the Campeche seeps and their MOX bacteria, with the goal of better understanding the evolutionary history and physiology of the MOX symbionts, revealing the mechanisms that might determine the specificity of the sponge-MOX association, comparing the genomic potential of the symbiotic MOX with that of free-living MOX, and tracing the incorporation of methane-derived carbon into sponge biomass.

## Materials and methods

### Sample collection

**Fig. 1:**
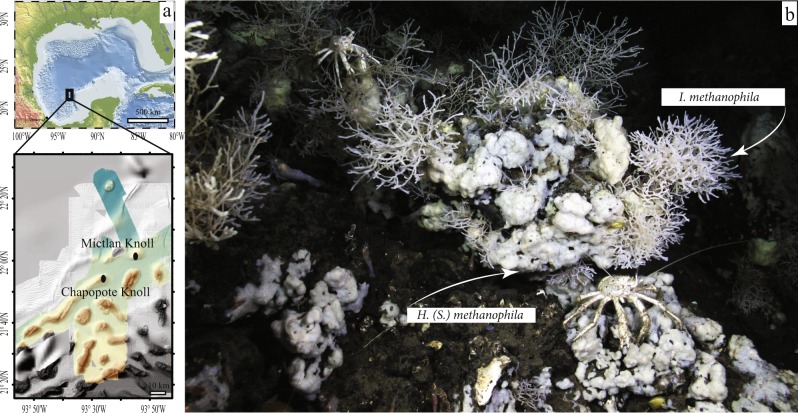
The encrusting sponge *Hymedesmia* (*Stylopus*) *methanophila* sp. nov. and the branching sponge *Iophon methanophila* sp. nov. colonize asphalt seeps at Campeche Knolls. **a** Geographic location of Campeche Knolls and the sponge collection sites, Chapopote and Mictlan. **b** MARUM-QUEST ROV image of *H*. (*S*.) *methanophila* and *I. methanophila* at Chapopote. Galatheid crabs graze on the sponges

Sponges were collected with the remotely-operated vehicle MARUM-QUEST during the RV Meteor M114-2 cruise to the Campeche Knolls in March 2015. The sponges were collected by placing asphalt pieces on which they grew in an insulated polypropylene “bio-box” to protect against temperature changes during the ascension of the ROV to warm surface waters (ascension time from seafloor to onboard ship ~2 h). We sampled two encrusting sponge individuals, one from Chapopote Knoll (21°54′ N; 93°26′ W, 2925 m water depth) and one from Mictlan Knoll (22°1′ N; 93°14′ W, 3106 m water depth), which are ~25 km apart from each other (Fig. 1). A third sponge individual with a branching morphology was collected from the same site at Chapopopote as the encrusting sponge. The sponges appeared healthy and intact before collection, with no evidence of tissue damage. A detailed description of the collection sites is available elsewhere [32]. Sponge distributions at Chapopote were estimated using mosaic mapping (see Supplementary Methods 4).

After ROV recovery, the asphalt pieces with the sponges were kept on board in the seawater from the “bio-box” at 4 °C. For metagenomic and metatranscriptomic analyses, subsamples from each sponge individual were fixed on board ~1 h after ROV recovery in RNAlater® (Sigma, Steinheim, Germany) according to the manufacturer’s instructions and stored at −80 °C. Subsamples for microscopy were fixed in 2% paraformaldehyde in 1x phosphate-buffered saline (PBS) for at most 12 h at 4 °C, rinsed three times in 1x PBS, and stored at 4 °C in 0.5x PBS/50% ethanol. Subsamples for transmission electron microscopy were fixed in PHEM buffer (PIPES, HEPES, EGTA and MgCl_2_, see ref. [34]). Samples for lipid biomarker analysis were flash-frozen in liquid nitrogen and stored at −80 °C.

### Fluorescence in situ hybridization (FISH)

The probe used in this study, MTC849 probe (5′-CGTTAGCTCCACCACTAAG-3′), was designed with ARB [35] to target the 16S rRNA gene sequences of the symbiotic MOX of both Campeche sponge species. This probe is a modification of MTC850 probe, designed to target Marine Methylotrophic Group (MMG) 2 MOX [36]. Apart from the symbiotic and MMG2 MOX, the MTC849 probe targets the closely related *Methylomonas* and *Methylomarinum* clades. The MTC849 oligonucleotide was double-labeled with Atto594 dye (Biomers, Ulm, Germany), and applied to 8 μm sections of sponge tissue using hybridization buffer with 20% formamide as described previously [37]. These hybridization conditions are assumed to ensure specificity, given the three mismatches that the MTC849 probe had to the 16S rRNA gene sequences of all other Campeche sponge bacteria [38].The general bacterial probe EUB338 [39] was used as a positive control and the NON338 probe was used as a control for background autofluorescence [40]. Photomicrographs were acquired with a Zeiss Axioplan 2 epifluorescence microscope (Zeiss, Jena, Germany) or with a confocal laser-scanning microscope (LSM 780, Carl Zeiss, Germany). Brightness and contrast of the images were adjusted with Adobe Photoshop (Adobe Systems, Inc., USA).

### Transmission electron microscopy (TEM)

Subsamples of the two encrusting and one branching sponge individuals were washed with 50 mM cacodylate buffer, post-fixed with 2% osmium tetroxide in the buffer for 1.5 h at 4 °C, and then washed with Milli-Q water. Following overnight staining in 0.5% uranyl acetate (Merck, Germany), the samples were washed with Milli-Q water, dehydrated in a graded ethanol series, and then transferred into propylene oxide (Sigma-Aldrich, Germany). The samples were infiltrated with Epon-812 resin (1:1 resin to propylene oxide) overnight and rinsed in this resin twice for 2 h. They were then transferred into fresh resin for 1 h and polymerized in embedding capsules at 60 °C for at least 48 h. Ultrathin (70 nm) sections were cut with an ultramicrotome (Leica EM UC7, Austria), mounted on pioloform coated grids, and contrasted with 2.5% uranyl acetate in ethanol for 20 min and subsequently, with Reynold’s lead citrate for 10 min. Ultrathin sections were imaged at 80 kV on a Tecnai G2 Spirit BioTwin transmission electron microscope (FEI Company, USA). We were able to produce high-quality micrographs of the two encrusting sponge individuals, but not of the branching sponge individual.

### DNA and RNA extraction and sequencing

We extracted DNA and RNA from the two encrusting sponge individuals and from two different branches of the same branching sponge individual. DNA and RNA were extracted in parallel with the AllPrep DNA/RNA Mini Kit (Qiagen, Hilden, Germany) according to the manufacturer’s recommendations, with an extra DNase I digestion step on RNA columns to eliminate contaminating DNA. DNA/RNA quality was assessed with the Agilent 2100 Bioanalyzer (Agilent, Santa Clara, USA). We were not able to extract RNA in sufficient amounts for transcriptomic analyses of the branching sponge individual. cDNA was synthesized with Ovation RNA-Seq System V2 (NuGen, San Carlos, CA, USA). Genomic DNA and cDNA libraries were generated with the DNA library prep kit for Illumina (BioLABS, Frankfurt am Main, Germany). All samples were sequenced on the Illumina HiSeq 2500 platform at the Max Planck Genome Centre (Cologne). For one subsample of the branching sponge, 41 million 150 bp paired-end metagenomic reads were generated. For the remaining three libraries, 12.5 million 250 bp paired-end metagenomic reads were generated, while the remaining 15.5–29.5 million were generated as 150 bp paired-end reads. In total, 30 and 38 million 100 bp paired-end cDNA reads were generated from the Mictlan and Chapopote encrusting sponge RNA extracts, respectively.

### Genome analyses

Individual metagenomes were assembled with IDBA-UD [41], following decontamination, quality filtering (QV = 2) and adapter-trimming with the BBDuk tool from the BBMap suite (Bushnell B, http://sourceforge.net/projects/bbmap/). Individual symbiont genomes were binned based on genome coverage, GC content and taxonomic affiliation using gbtools (Supplementary Figure S1) [42]. These bins were reassembled with Spades V3.10 [43, 44], using a maximum k-mer length of 127, following re-mapping of Illumina reads to the bins using BBMap with 0.98 minimum identity. Following the manual removal of contigs shorter than 800 bp and contamination screening, quality metrics were calculated with Quast [45] and CheckM [46]. Symbiont draft genomes and transcriptome reads were deposited in NCBI under accession numbers PRJNA475438 and PRJNA475442. Downstream genome analyses are summarized in Supplementary Methods S1.

### Transcriptome analyses

Adapters and ribosomal RNA genes were removed from transcriptome reads with BBDuk. Transcriptome reads were mapped to the individual methanotroph genome assemblies with BBMap (minimum identity value of 0.98). Mapped reads were assigned to genomic features with featureCounts [47]. Relative RNA levels were estimated with transcripts per million (TPM) normalization [48]. The metatranscriptomes were assembled with Trinity [49] and the transcripts were quantified with an align_and_estimate_abundance.pl script from the Trinity package, using RSEM quantification method [50].

### Phylogenomics

Phylogenomic reconstructions were performed with phylogenomics-tools scripts (10.5281/zenodo.46122). Marker proteins that are universally conserved across the bacterial domain were extracted from genomes using AMPHORA2 [51]. Eighteen single-copy markers that were present in all genomes analyzed in this study were used for alignment with MUSCLE [52]. The marker alignments were concatenated into a single partitioned alignment. Poorly aligned or misaligned regions were removed from the alignments. The maximum likelihood tree was calculated with MEGA7 [53] using the LG model [54].

### Lipid biomarker analysis

Lipid biomarkers were extracted from one individual of branching and one individual of encrusting sponge (both collected at Chapopote). Phospholipid fatty acids were hydrolyzed by saponification with 6% KOH in methanol. Sterols were extracted with dichloromethane:methanol (3:1) three times. The resulting total lipid extract and the saponification extract were combined. The hydrolyzed free fatty acid salts were released from the aqueous phase by adding HCl until pH 2. Then, the combined extracts were separated by solid phase column chromatography into four fractions. The resulting fatty acids and the alcohols were analyzed on a Thermo Electron Trace DSQ II coupled gas-chromatograph-mass spectrometer (GC-MS) for quantification and identification. The GC-MS was equipped with a 30 m HP-5 MS UI fused silica capillary column (0.25 mm i.d., 0.25 µm film thickness). The carrier gas was helium. The GC temperature program used for both fractions was as follows: 60 °C (1 min), from 60 to 150 °C at 10°/min, from 150 to 325 °C at 4 °C/min, 25 min isothermal. Identification of compounds was based on retention times and published mass spectral data. Double bond positions of unsaturated fatty acids were identified by the addition of a dimethyl disulfide (DMDS) adduct to an aliquot of the saponified fatty acid fraction [55]. Further, compound-specific carbon stable isotope compositions of fatty acids and sterols were measured on a gas chromatograph (Agilent 6890) coupled with a Thermo Finnigan Combustion III interface to a Finnigan Delta Plus XL isotope ratio mass spectrometer (GC-IRMS). The GC conditions were identical to those mentioned above for GC-MS analyses.

## Results and Discussion

### Novel sponge species are abundant at Chapopote

Morphological analyses of both sponge morphotypes revealed that these have not yet been described. We name them here *Hymedesmia* (*Stylopus*) *methanophila* sp. nov. and *Iophon methanophila* sp. nov. (order Poecilosclerida). These belong to two different genera and are only distantly related to each other (based on 90% identity of their cytochrome c oxidase subunit I (*COI*) gene sequences). A full description of the morphology and phylogeny of these two sponge species is available in Supplementary File SF1.

At Chapopote and Mictlan, *H*. (*S*.) *methanophila* and *I. methanophila* occurred on fragmented asphalt accumulations next to sites of active seepage, characterized by gas and oil effusions and darkly colored substrates (Supplementary Figure S2). *I. methanophila* individuals were often observed growing on the tubes of tubeworms. Based on our mosaic mapping of the Chapopote site, we estimate that the sponges colonized ~30% of the hard substrates in areas of active seepage (Supplementary Figure S2). We often observed grazers in association with the sponges, in particular, galatheid crabs (Fig. 1). These observations, together with observations of similar sponge abundances during a 2005 cruise to Chapopote [56], suggest that these sponges have made up a considerable part of the biomass at Chapopote for over twelve years, and are thus likely to influence the composition of the seep community and contribute to its food web.

### Campeche sponges host high abundances of methane-oxidizing bacteria

Analyses of the microbial communities hosted by *H*. (*S*.) *methanophila* and *I. methanophila* revealed that their dominant members were MOX (35.9–67.6% of the microbial community; these values and the following in this paragraph are based on relative 16S rRNA read frequencies in the metagenomes, as well as the frequencies of metagenomic reads that mapped to the MOX genomes, see Supplementary Methods and Table 1). In both host species, Proteobacteria made up the vast majority of the microbial community (94.8–99.9%), and included chemoautotrophic sulfur-oxidizing bacteria from the SUP05 clade (4.6–20.2%) and hydrocarbon-degrading *Cycloclasticus* symbionts (6.0–8.4%), which have been described elsewhere [33]. Such high abundances of Proteobacteria have not been commonly found in HMA sponges. Most HMA sponges harbor highly diverse microbial communities that are often dominated by the bacterial phyla Chloroflexi, Acidobacteria, and Actinobacteria [57].

**Table 1:**
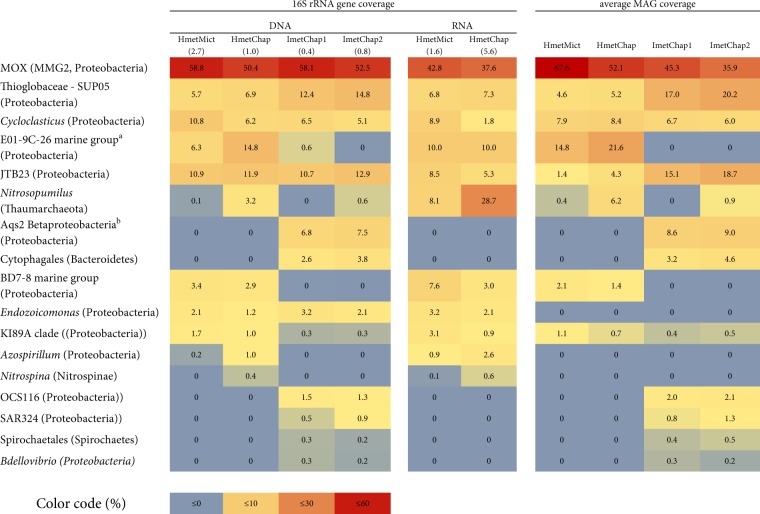
Relative abundance and phylogenetic assignment of sponge-associated bacteria based on metagenomic and metatranscriptomic coverage of their 16S rRNA gene sequences and the coverage of metagenome assembled genomes (MAGs)

Classifications at the Phylum level are mentioned in parentheses. The 16S rRNA sequences of the MOX symbionts were identical within each host species, and differed slightly, but distinctly between the two host species (97.8% identity). The following names are abbreviated: *H*. (*S*.) *methanophila* from Mictlan (HmetMict) and Chapopote (HmetChap); *I*. *methanophila* from Chapopote subsamples 1 and 2 (ImetChap1 and ImetChap2); methane-oxidizing bacteria (MOX); marine methylotrophic group 2 (MMG2). Numbers in parentheses within the column headers represent the ratio between the MOX 16S and the host’s 18S rRNA gene coverage

^a^E01-9C-26 marine group is a monophyletic ‘sponge-enriched’ gammaproteobacterial clade

^b^Aqs2 symbiont is a monophyletic ‘sponge-enriched’ clade of Proteobacteria, which includes symbionts of the low microbial abundance sponges *Amphimedon queenslandica* and *Crambe crambe*

**Fig. 2:**
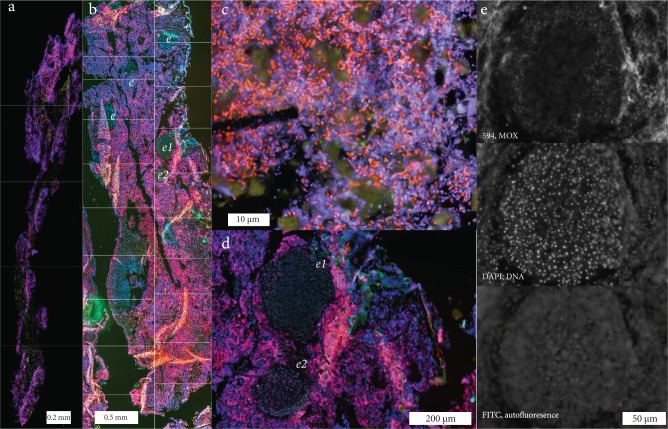
Fluorescence in situ hybridization (FISH) images of the symbiotic methane-oxidizing bacteria (MOX) in Campeche sponges. FISH was performed with a probe specific to the symbiotic MOX on 10 μm thick sections of *I. methanophila* and *H*. (*S*.) *methanophila*. Colors: MOX symbionts, magenta; DNA (DAPI staining), blue; autofluorescence at ~520 nm with ~490 nm excitation (FITC filter), green. **a** Overview of *I. methanophila*. The image is a mosaic of five aligned images (lines mark borders between the images). **b** Overview of *H*. (*S*.) *methanophila*. The image is a mosaic of twenty aligned images (lines mark borders between the images, *e*: embryos in various developmental stages. **c** MOX symbionts are abundant in the mesohyl of *I. methanophila*. **d** Detail of embryos labeled *e1* and *e2* in panel (**b**). **e** FISH images of embryo labeled *e2* in panel (**b**) using gray intensity representation to distinguish FISH signal of MOX symbionts (top panel) from DAPI staining (middle panel) and autofluorescence (bottom panel). Images from the mosaics, additional images of MOX in tissues of *H*. (*S*.) *methanophila* embryos and 3-dimensional z-stack reconstructions are available at https://figshare.com/projects/Fueled_by_methane_Deep-sea_sponges_from_asphalt_seeps_gain_their_nutrition_from_methane-oxidizing_symbionts/23020

Although the low phylogenetic diversity of the microbial community in Campeche sponges is typical of LMA hosts, fluorescence in situ hybridization (FISH) revealed that the abundances of bacteria were more typical of HMA sponges [5] (Fig. 2). FISH with a probe specific to the MOX 16S rRNA sequences in both host species revealed that these bacteria were present in high numbers in the sponge mesohyl (the gelatinous matrix within a sponge that fills the space between its external and internal cell layers). Although the Campeche sponges belong to an order (Poecilosclerida) previously described as consisting of only LMA species, they have traits typical of both LMA sponges (low phylogenetic diversity) and HMA sponges (high symbiont abundances). They thus represent an exception to the described dichotomy between HMA and LMA sponges.

**Fig. 3:**
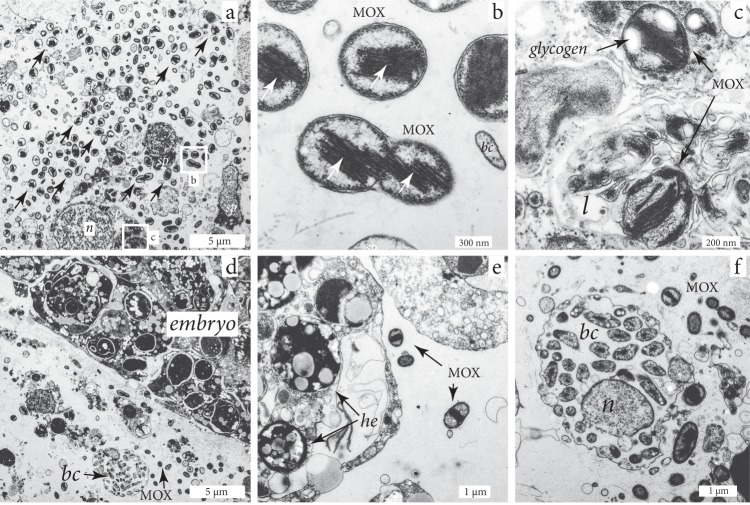
Transmission electron microscopy images of *H*. (*S*.) *methanophila*. **a** Symbiotic methane-oxidizing bacteria (MOX) are abundant in the mesohyl, particularly in regions close to the choanocyte chambers (chambers not visible in image), *sp* = sponge cells, *n* = nucleus. **b** High-resolution image of white box labeled b from panel (**a**), showing intracellular membranes (arrows) typical for MOX. The MOX at the lower half of the image are dividing; *bc* = bacteria with a different morphology than the MOX symbionts. **c** MOX symbiont in a lysosome of an amebocyte, *l* = lysosome. **d** Mesohyl with an embryo surrounded by symbiotic MOX, and a bacteriocyte containing bacteria with a different morphology than the symbiotic MOX (*bc*). **e** MOX symbionts within an embryo. *he* = heterogeneous yolk. **f** A bacteriocyte containing bacteria with a different morphology than the MOX symbionts. *bc* = bacteria; *n* = nucleus

Transmission electron microscopy (TEM) of *H*. (*S*.) *methanophila* revealed that their mesohyl was colonized by a bacterial morphotype characterized by (i) a coccoid shape and intracytoplasmic membrane stacks typical of MOX bacteria; (ii) sizes of 500–1300 nm length and 500–900 nm width, and (iii) electron-lucent granules typical of storage compounds, most likely glycogen (Fig. 3a, Supplementary Note 1). The distribution pattern of these morphotypes was similar to that of the MOX bacteria identified with FISH, indicating that we could identify the MOX symbiont of *H. (S.) methanophila* with TEM based on its characteristic ultrastructure. We regularly observed MOX in stages of division, indicating that they were actively growing in the sponge matrix (Fig. 3b). The MOX were generally extracellular, but we occasionally found them within host vacuoles in different stages of degradation (Fig. 3c), indicating that the sponge cells engulf and digest these symbionts. Bacteria with a very different morphology than that of the MOX symbionts occurred in what looked like specialized bacteriocyte cells described from other sponge species [58, 59], but we never observed MOX in these host cells (Fig. 3f). The symbiotic MOX were most abundant in mesohyl regions near the choanocyte chambers (Fig. 3a). This indicates that the symbiotic MOX benefit from being close to the flagellated choanocytes, most likely because these host cells pump the methane- and oxygen-containing seawater from the seep environment into the sponge matrix, and thus provide the symbionts with the reductants and oxidants they need for their energy and carbon metabolism.

### Specificity of the sponge-MOX association

The comparison of the MOX symbiont genomes from the two Campeche sponge species suggests that these are specific to their hosts. The two *H*. (*S*.) *methanophila* individuals that were collected 25 km apart at Chapopote and Mictlan, hosted nearly identical MOX genotypes (99.6 ± 0.9% average nucleotide identity (ANI)). Only one *I. methanophila* individual could be collected from the Campeche seeps (at Chapopote), but ANI values of the MOX symbiont genomes from two different branches of this individual were nearly identical (100 ± 0.6%). Comparison of the MOX symbiont genomes from the sympatric *H*. (*S*.) *methanophila* and *I. methanophila* individuals, which were collected from the same asphalt piece at Chapopote, revealed that these differed considerably from each other (93.6 ± 2.5% ANI), and can be considered to belong to two different species (ANI < 95%, [59]). These results suggest that each sponge species hosts a specific MOX genotype, and imply the presence of recognition and selection mechanisms that underlie the potential specificity of this symbiosis.

### The symbiotic MOX belong to the Marine Methylotrophic Group 2 clade

**Fig. 4:**
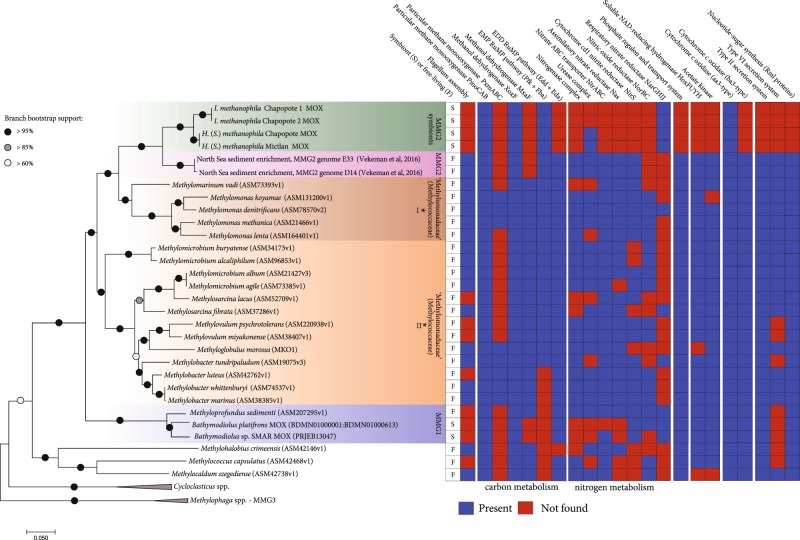
Phylogenomic tree and metabolic repertoire of the sponge MOX symbionts and related bacteria (45 sequences total). The sponge MOX symbionts and the two free-living MOX from North Sea sediment enrichments (provided by B. Vekeman) are currently the only genomes available for the MMG (Marine Methylotrophic Group) 2, although 16S rRNA data indicates that they are widespread (see Supplementary Figure S3 16S rRNA tree). Eighteen single-copy markers as defined in the AMPHORA core bacterial phylogenetic marker database were used in the analysis. The tree is drawn to scale, with branch lengths representing the number of substitutions per site. The percentage of trees in which the associated taxa clustered together was determined based on 100 bootstrap resamples. The analysis included 3706 positions. *These clades were formerly included in the family Methylococcaceae, and recently placed together with the MMG1 and MMG2 clades within the family ‘Methylomonadaceae’ (order Methylococcales), based on the Genome Taxonomy Database

Phylogenomic reconstruction of eighteen marker proteins, as well as the phylogenies of 16S rRNA and *pmoA* (particulate methane monooxygenase) genes, revealed that the sponge-associated MOX belong to the Marine Methylotrophic Group (MMG) 2 clade (also known as deep-sea clade 2) within the family ‘Methylomonadaceae’, order Methylococcales (Fig. 4, Supplementary Figures S3 and S4) [61]. To date, there are no pure cultures of bacteria from the MMG2 clade, but recently the genomes of two MMG2 MOX enriched from North Sea sandy sediments with methane as the sole carbon and energy source were sequenced [62]. Together with the Campeche MOX symbionts, these are currently the only genomes that have been sequenced from the MMG2 clade.

Interestingly, MMG2 bacteria have been reported from other marine hosts based on 16S rRNA and *pmoA* gene sequencing, although their abundances appear to generally be considerably lower than in the Campeche sponges (Supplementary Figures S3 and S4). MMG2-related sequences were described from ciliates collected at methane seeps along the eastern Pacific coast (1.7–9.7% of the ciliate bacterial community based on 16S rRNA tag sequencing) [63], and the squat lobster *Shinkaia crosnieri* from hydrothermal vents off Japan (1.7–12.0% of 16S rRNA clones), [64]. MMG2 sequences were also found in two sponge species: (i) an unidentified poecilosclerid sponge from seeps in the Gulf of Mexico that is morphologically distinct from our samples [28], and (ii) *Cladorhiza methanophila* from mud volcanoes off Barbados (16–25% based on 16S rRNA tag sequencing [25]). MOX belonging to the MMG2 clade were also found in a *Sibolginum* cf. *poseidoni* tubeworm from a mud volcano in the Northeast Atlantic (the nine 16S rRNA clones sequenced from the tubeworm all belonged to MMG2 [65]).

Common to all these hosts is that they occur in methane-rich environments, such as mud volcanoes, seeps, and hydrothermal vents, where free-living MMG2 bacteria are known to be abundant in both the sediment and the water column [36]. Given the broad range of hosts associated with bacteria from the MMG2 clade, from protists to animals from different phyla, it is highly likely that these associations were established multiple times independently of each other. Moreover, the MOX bacterial partners in these associations are phylogenetically distinct from those of the deep-sea bathymodiolin mussels, which belong to the MMG1 clade (Fig. 4 and Supplementary Figures S3 and S4) [66]. Our study thus contributes to revealing the previously unrecognized diversity of methanotrophic associations on both the bacterial and host side. This diversity suggests that there are strong selective advantages for both partners in establishing these beneficial associations in methane-rich environments. Revealing the factors that contribute to these selective advantages, and distinguishing these from the mechanisms behind the broad host promiscuity in MMG1 MOX and the apparent specificity of MMG2 MOX to bathymodiolin mussels, will contribute to understanding the ecological and evolutionary requirements for establishing these symbioses.

### The symbiotic MOX may be vertically transmitted

We found evidence for vertical transmission of the symbiotic MOX from one generation to the next. Embryos in the matrix of both *H*. (*S*.) *methanophila* individuals contained MOX symbionts based on FISH and TEM analyses (Figs. 2 and 3; Supplementary Figure S5). The symbiotic MOX were observed in the matrix between the embryo cells, but never inside of sponge cells (Fig. 3e). MOX were also found between follicle-like cells surrounding the egg, as well as on the periphery of follicle-like cells (Supplementary Figure S5). In the seep sponge *Cladorhiza methanophila*, bacteria with features typical of methane-oxidizing bacteria were also observed in embryos with TEM, indicating that symbionts are transmitted vertically in this sponge species as well [67].

### Genome reduction in the symbiotic MOX

**Fig. 5:**
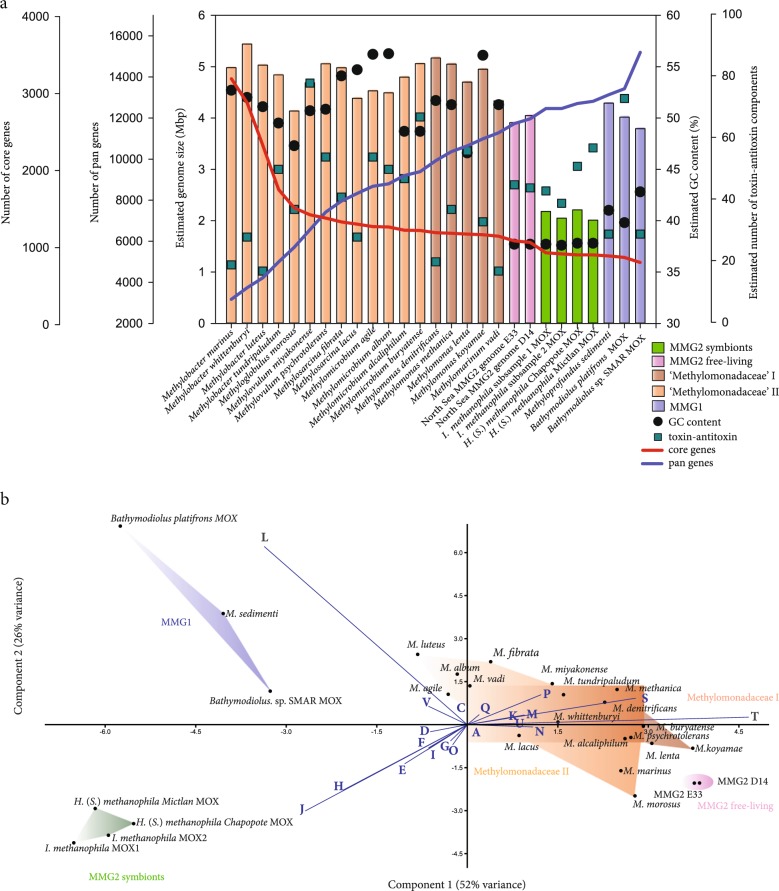
Comparison of genomes from the sponge symbionts and other gammaproteobacterial methane-oxidizing bacteria (MOX). **a** Distribution of estimated genome sizes, guanine-cytosine (GC) content and the estimated number of toxin-antitoxin components in genomes of methane-oxidizing bacteria (MOX). The blue and red lines show pan genomes (blue) versus core genome (red) development plots (obtained by iteratively adding one genome at a time to the comparison in the defined order (starting with the first genome from the left). Comparative genomic analysis led to a pan-genome estimate of 16476 coding sequences, of which 1050 formed the core genome. The gradual change in the slope of the pan-genome development curve for the MMG 2 symbionts suggests that this clade is sufficiently sampled. 163 genes were subtracted from the core genome of ‘Methylomonadaceae’ after addition of the sponge symbionts. Mbp = million base pairs. **b** Principal component analysis based on the relative abundance of clusters of orthologous groups (COGs) encoded by symbiotic and free-living MOX. The following COG abbreviations are shown: [D] cell cycle control, cell division, chromosome partitioning, [M] cell wall/membrane/envelope biogenesis, [N] cell motility, [O] post-translational modification, protein turnover, and chaperones, [T] signal transduction mechanisms, [U] intracellular trafficking, secretion, and vesicular transport, [V] defense mechanisms, [A] RNA processing and modification, [J] translation, ribosomal structure and biogenesis, [K] transcription, [L] replication, recombination and repair, [C] energy production and conversion, [E] amino acid transport and metabolism, [F] nucleotide transport and metabolism, [G] carbohydrate transport and metabolism, [H] coenzyme transport and metabolism, [I] lipid transport and metabolism, [P] inorganic ion transport and metabolism, [Q] secondary metabolites biosynthesis, transport, and catabolism, [S] function unknown

Additional support for our conclusion that the Campeche sponge MOX symbionts are vertically transmitted is provided by the comparison of their genome sizes to those of free-living MOX bacteria. The estimated genome sizes of the Campeche MOX symbionts were between 2.0 and 2.2 Mbp, and thus considerably reduced in comparison to the 3.9 and 4.0 Mbp genomes of their close relatives from North Sea sediment enrichments (Fig. 5a, Supplementary Table S1). The genomes of the sponge MOX symbionts were also reduced in comparison to those of cultivated ‘Methylomonadaceae’ (4.5–5.2 Mbp), although some free-living ‘Methylomonadaceae’ may have similarly small genomes, e.g., Methylococcales bacterium OPU3_GD_OMZ from a marine metagenome [68]. Given that the sponge-associated MOX genomes were highly complete (94.6–96.5%), it is unlikely that we underestimated their sizes due to incomplete binning (Supplementary Table S1). As genome reduction is typical for vertically transmitted symbionts, the small genome sizes of the Campeche sponge MOX symbionts may be a result of accelerated genome evolution through vertical transmission [69–71]. The very low guanine + cytosine (GC) content of the symbiont genomes (37.7 ± 0.1% versus 51 ± 4% in cultivated ‘Methylomonadaceae’) may also be a result of vertical transmission, as known from many other vertically transmitted symbionts Fig. 5a) [72]. However, the closest free-living relatives of the sponge symbionts, the North Sea bacteria from sediment enrichments (Fig. 4), have similarly low GC contents of 37.7%, indicating that other factors, such as energetic constraints, may have played a role in the genome evolution of this clade [73, 74].

### Optimization of methane assimilation in sponge symbionts

To better understand the functional adaptations of the sponge MOX to their symbiotic lifestyle, we compared their genomes to those of free-living MOX within the ‘Methylomonadaceae’ as well as the MOX symbionts of bathymodiolin mussels (Figs. 4 and 5). We identified 1050 genes in the core genome of the free-living ‘Methylomonadaceae’, 163 of which were not found in the sponge MOX symbionts (Fig. 5a, Supplementary Table 3a). Comparative genomics revealed that the functional repertoire of the sponge MOX symbionts for the assimilation of methane and other carbon compounds was reduced in comparison to free-living bacteria within the ‘Methylomonadaceae’ (Figs. 4, 5b, Supplementary Figure S6).

**Fig. 6:**
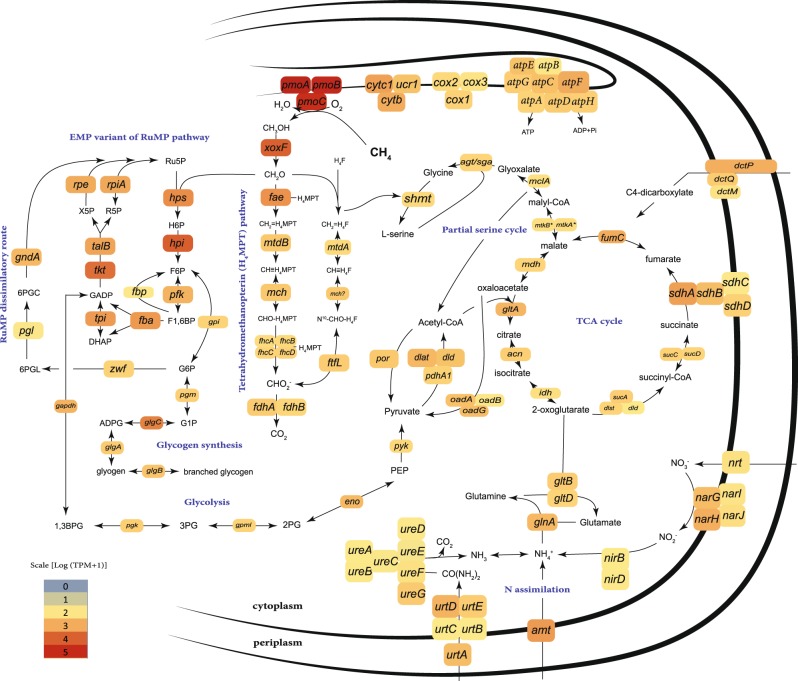
Central carbon and nitrogen metabolism in the sponge MOX symbionts. The reconstruction is based on the genomes of *I. methanophila* and *H*. (*S*.) *methanophila* symbionts and on the average expression in the two transcriptomes of *H*. (*S*.) *methanophila* symbionts. Boxes represent enzyme subunits and the abbreviations represent the genes that encode the respective subunit. Boxes are colored according to the expression value of the gene. The following genes are abbreviated: *pmoABC*, particulate methane monooxygenase subunits A, B and C; *xoxF*, methanol dehydrogenase; *fae*, formaldehyde activating enzyme; *mtdB*, methylene tetrahydromethanopterin dehydrogenase; *mch*, methenyltetrahydromethanopterin cyclohydrolase; *fhcABCD*, formyltransferase/hydrolase complex; *fdhAB*, formate dehydrogenase subunits alpha and beta; *mtdA*, methylene tetrahydrofolate/methylene tetrahydromethanopterin dehydrogenase; *ftfL*, formate-tetrahydrofolate ligase; *hps*, 3-hexulose-6-phosphate synthase; *hpi*, 6-phospho-3-hexuloisomerase; *pfk*, pyrophosphate-dependent phosphofructokinase; *fbp*, fructose-1,6-bisphosphatase; *fba*, fructose-bisphosphate aldolase; *tpi*, triosephosphate isomerase; *tkt*, transketolase; *talB*, transaldolase; *rpe*, ribulose-phosphate 3-epimerase; *rpiA*, ribose-5-phosphate isomerase; *gpi*, glucose-6-phosphate isomerase; *zwf*, glucose-6-phosphate 1-dehydrogenase; *pgl*, 6-phosphogluconolactonase; *gndA*, 6-phosphogluconate dehydrogenase; *gapdh*, glyceraldehyde 3-phosphate dehydrogenase; *pgk*, phosphoglycerate kinase; *gpml*, 2,3-bisphosphoglycerate-independent phosphoglycerate mutase; *eno*, enolase; *pyk*, pyruvate kinase; *por*, pyruvate-flavodoxin oxidoreductase; *dlat*, acetyltransferase component of pyruvate dehydrogenase complex; *dld*; dihydrolipoamide dehydrogenase of pyruvate or 2-oxoglutarate dehydrogenase complexes; *pdhA1*, pyruvate dehydrogenase E1 component subunit alpha; *oadABG*, oxaloacetate decarboxylase, alpha, beta and gamma chains; *pgm*, phosphoglucomutase; *glgC*, glucose-1-phosphate adenylyltransferase; *glgA*, glycogen synthase; *glgB*, 1,4-alpha-glucan branching enzyme; *smht*, serine hydroxymethyltransferase; *agt*/*sgaA*, serine-glyoxylate aminotransferase; *mclA*, malyl-CoA lyase; *mtkAB*, malate thiokinase, alpha and beta subunits; *mdh*, malate dehydrogenase; *gltA*, citrate synthase; *acn*, aconitase; *idh*, isocitrate dehydrogenase; *sucA*, 2-oxoglutarate dehydrogenase E1 component; *dlst*, dihydrolipoyllysine-residue succinyltransferase component of 2-oxoglutarate dehydrogenase complex; *sucCD*, succinate-CoA ligase subunits beta and alpha; *sdhABCD*, succinate dehydrogenase complex subunits; *fumC*, fumarate hydratase; *dctMPQ*, C4-dicarboxylate TRAP transporter subunits; *gltDB*, glutamate synthase, large and small chains; *glnA*, glutamine synthetase; *amt*, ammonium transporter; *nrt*, nitrate transporter; *nirBD*, assimilatory nitrite reductase small and large subunits; *narGHIJ*, respiratory nitrate reductase, alpha-gamma subunits; *urtABCDE*, urea ABC transport system subunits; *ureABCDEFG*, urease subunits; *atpABCDEFGH*, subunits of the membrane-bound ATP synthase; *cox123*, cytochrome c oxidase subunit I-III; *cytc1*, cytochrome c-1, cytochrome b-c1 complex; *urc1*, cytochrome b-c1 complex subunit 1; *cytb*, cytochrome b, cytochrome b-c1 complex

Similar to other ‘Methylomonadaceae’, the sponge MOX symbionts are type I methanotrophs: The ribulose monophosphate (RuMP) cycle appears to be their sole pathway for methane incorporation, as key enzymes for the assimilatory serine cycle, such as hydroxypyruvate reductase, were not found [75]. Genes not found in the sponge MOX included those encoding the Entner–Doudoroff (EDD) pathway enzymes, phosphogluconate dehydratase (Edd) and 2-dehydro-3-deoxy-phosphogluconate aldolase (Eda), which play a role in methane carbon assimilation via the RuMP pathway [76]. The sponge MOX symbionts appear to assimilate single carbon compounds only via the highly efficient Embden–Meyerhof–Parnas (EMP) variant of the RuMP cycle, based on the presence and substantial expression of the genes encoding fructose-bisphosphate aldolase (*fba*) and pyrophosphate-dependent phosphofructokinase (*pfk*) [76] (Fig. 6). The sponge MOX symbionts may, therefore, be more efficient in using single carbon compounds than the methane-oxidizing symbionts of bathymodiolin mussels, which only employ the less efficient EDD variant of the RuMP pathway [76, 77]. This suggests that the symbiotic MOX of sponges are able to provide nutrition to their hosts at lower methane concentrations than the mussel symbionts. This hypothesis is supported by our mapping analyses of the seafloor at Chapopote, as sponges were often situated further from hotspots of active gas and oil seepage than the mussels (Supplementary Figure S2).

The symbiotic MOX appear to use a minimal suite of enzymes needed for methane assimilation. As opposed to most sequenced, free-living MOX, in which two variants of methanol dehydrogenase, the lanthanide-dependent (XoxF) and the calcium-dependent (MxaF) methanol dehydrogenase co-occur, only XoxF was found in the sponge symbionts (Fig. 4). Furthermore, only one *pmoCAB* operon encoded the methane monooxygenase, while the *pxmABC* operon was not found (Fig. 4). Despite this genetic minimalism in genes involved in methane oxidation, these genes, together with the RuMP pathway, were highly expressed by the symbionts (Fig. 6). Moreover, these genes were among the top 1% of the most well-expressed transcripts in the metatranscriptomes, which included reads that mapped to both the host and the symbionts. This suggests that methane assimilation by the symbiotic MOX was among the most active metabolic processes in the sponge holobiont.

The sponge MOX symbionts may be able to use other sources of carbon besides methane. Their genomes contained the genes for the TCA cycle, glycogen synthesis and degradation, as well as the import of multicarbon substrates via C4-dicarboxylate tripartite ATP-independent periplasmic (TRAP) transporters (Fig. 6, Supplementary Note 1). Moreover, most of these genes were expressed (Fig. 6). This suggests that similar to the mussel MOX symbionts, which also may use other carbon sources besides methane [77], the sponge MOX symbiont have evolved mechanisms to deal with fluctuations in methane availability. Indeed, our TEM observations of electron-lucent granules typical of carbon storage compounds in the MOX sponge symbionts supports our assumption that glycogen may be used to buffer against periods of methane deprivation.

### Reductive evolution and adaptation to the symbiosis

Comparative analyses of clusters of orthologous groups (COGs) in the sponge MOX with those of free-living ‘Methylomonadaceae’, as well as the MOX symbionts of bathymodiolin mussels revealed that physical and biotic interactions with the environment appear to have shaped the pan-genome of free-living MOX. In these MOX, functions such as “cell motility”, “inorganic ion transport and metabolism”, “signal transduction mechanisms”, “membrane biogenesis”, and “function unknown” (this diverse group includes toxins, type VI secretion system, antibiotic resistance, phage and plasmid proteins) were enriched in comparison to the sponge and mussel symbionts (Fig. 5b). The COGs “translation, ribosomal structure and biogenesis”, “coenzyme transport and metabolism” and “amino acid transport and metabolism” were enriched in the sponge MOX symbionts, suggesting that their metabolic repertoire beyond these housekeeping functions is limited. Large numbers of transposases and integrases resulted in the enrichment of the COG “replication, recombination and repair” in the MMG1 clade, which comprises symbionts of bathymodiolin mussels and a single free-living bacterium *M. sedimenti*. Expansion of transposable elements is more common in symbionts that have recently transitioned to an obligate, host-associated lifestyle, than in symbionts that have associated with their hosts over long evolutionary time periods [68, 69, 78, 79]. The sponge symbioses, therefore, may have pre-dated those of mussels. However, this is highly speculative, as the free-living MOX *M. sedimenti* also has high numbers of transposable elements. The only genes that were shared between the sponge and mussel symbionts, yet not present in most free-living ‘Methylomonadaceae’, were the *narGHIJ* genes, encoding enzymes for nitrate respiration (Supplementary Note 2). Thus, there appears to be little convergence in the mechanisms the sponge and mussel symbionts have evolved to establish and maintain associations with their hosts.

Analysis of the 163 core proteins that were unique to the free-living ‘Methylomonadaceae’ revealed that the sponge symbionts appear to lack the following functions: (i) nutrient uptake (nitrate ABC transporter NrtABC, the Pho regulon and phosphate transport system); (ii) secondary metabolite production and secretion (Rml proteins that catalyze synthesis of nucleotide/polyketide sugars and Gsp building blocks of the type 2 secretion system); and (iii) adaptation to hypoxia, based on the absence of the high-affinity ba3-type cytochrome oxidase [80] (Fig. 4, Supplementary Table 3a). Further indications that the sponge symbionts are not well adapted to hypoxia was the apparent lack of genes encoding the soluble NAD-reducing hydrogenase and acetate kinase, which catalyze fermentation of methane-derived products in many other MOX [76] (Fig. 4). Some genes and pathways involved in nitrogen metabolism also appear to be lacking in the sponge symbionts. Unlike most free-living MOX, the sponge symbionts may be incapable of nitrogen fixation and dissimilatory nitrite reduction to nitrous oxide (Fig. 4). As suggested previously for sulfur-oxidizing symbionts [81], a divergent respiratory nitrate reductase NarGHIJ, uncommon in most MOX, may play a role in non-canonical nitrate assimilation in the sponge MOX symbionts, which appear to lack the assimilatory nitrate reductase Nas (Supplementary Note 2). Altogether, these findings suggest a reduced functional repertoire of the sponge symbionts, which may have been shaped by (i) adaptation of the sponge MOX to the chemical environment within the host milieu, (ii) metabolic optimization via reduction of functional redundancy, and (iii) a very limited range of biotic interactions with free-living microorganisms. The reduction of such a wide array of functions that appear to be essential for a free-living lifestyle suggests that the sponge MOX may have entered the ‘rabbit hole’ of obligatory symbiosis, and are no longer able to actively grow outside of their host.

### Functional homogeneity between the symbiotic MOX from the two sponge species

Comparative genomics revealed that the MOX symbionts from the two sponge species were similar in most of their core metabolic pathways. In total, 1042 genes were common to their core genome, while 372 and 357 genes were unique to *H*. (*S*.) *methanophila* and *I*. *methanophila* symbionts, respectively (Supplementary Figure S6). The main fraction of the genomic content unique to each symbiont consisted of poorly-annotated open reading frames (ORFs), primarily including mobile elements and phage-derived sequences, often stabilized by toxin-antitoxin systems [82]. These toxin-antitoxin systems are abundant in the genomes of all sequenced aerobic methanotrophs and account for a significant part of their genomic variability (Fig. 5a). Only three sets of genes with well-described metabolic functions were present in the genomes of the *H*. (*S*.) *methanophila* MOX, but appear to be absent in those of the *I*. *methanophila* MOX: (i) genes encoding a urease and urea transporter, (ii) a gene encoding a C4-dicarboxylate transporter, and (iii) a secondary metabolite synthesis cluster, most likely an aryl polyene of unknown function [83] (Supplementary Figure S7). No genes with a well-described metabolic function were found that were unique to the *I. methanophila* MOX in comparison to the *H*. (*S*.) *methanophila* MOX.

### Eukaryote-like proteins are encoded in the genomes of the sponge MOX

The genomes of the sponge MOX symbionts contained 823 genes that were unique to the symbionts from both sponge species, and not present in free-living relatives (97 of which belong the sponge MOX symbiont core genome, see Supplementary Note 3 for more details). Some of these symbiont-specific sequences were ORFs as large as 14.6 kb, and contained one or more eukaryotic-like domains (ELD), such as leucine-rich repeats, cadherin-like domains, and bacterial immunoglobulin-like domains (Supplementary Table S3, Supplementary Figure S8). ORFs with multiple ELDs are well-known from intracellular bacterial pathogens and have also been regularly found in the bacterial metagenomes of other sponge species, where they encode eukaryote-like proteins (ELPs), which are likely involved in symbiotic interactions with their hosts (e.g., ref. [84]). In the MOX symbionts of *H*. (S) *methanophila*, some of the ELPs contained autotransporter beta-domains. These are known from many gram-negative bacteria, and encode a protein involved in forming a pore through the outer membrane [85]. This suggests that the ELPs could be secreted, providing further evidence for their potential role in symbiont–host interactions. BLAST analysis of the sponge MOX symbiont ELPs against the NCBI database resulted in best hits to the ELPs in the *Cycloclasticus* symbionts of the Campeche sponges (e.g., ORU94421.1, 98% identity and 78% coverage compared to the 14.6 kb ORF from the *H*. (*S*.) *methanophila* MOX). The similarity of ELPs from symbionts that inhabit the same host but belong to very distant bacterial lineages suggests the convergent evolution of these putative symbiosis factors.

### The symbiotic MOX appear to be the primary source of sponge carbon

Given the high abundances of MOX symbionts in both Campeche sponge species, we hypothesized that they play an important role in their nutrition. To test this hypothesis, we analyzed phospholipid fatty acids (PLFAs) and sterols in the sponges (see Supplementary Note 4 for more details). Monounsaturated fatty acids known to be specific to MOX [86], comprised 27% of all lipids of *H*. (*S*.) *methanophila* and 14% of all lipids in *I. methanophila* individuals, confirming high abundances of symbiotic MOX in both host species (Table 2). Carbon isotopic signatures of these fatty acids reflected those of Campeche methane (δ^13^C_methane_ was between −50 and −45‰ [32], δ^13^C_SpongeMOXfattyacids_ was between −51 and −44‰, Supplementary Table S3). This suggests that in contrast to cultivated type I methanotrophs [87, 88], the sponge MOX symbionts, as well as the mussel symbionts [86], do not fractionate methane carbon during the biosynthesis of their fatty acids, possibly due to periodic methane limitation.

**Table 2:** Relative composition of lipid biomarkers and their average δ^13^C values in sponge tissue

lipid biomarker sources	*H*. (*S*.) *methanophila*		*I. methanophila*	
	% of all lipids	δ^13^C (‰) (av.)	% of all lipids	δ^13^C (‰) (av.)
MOX (MUFAs)	27	−46	14	−50
Sponge (demospongic acids, MUFAs)	28	−47	39	−43
Sponge (sterols)	29	−41	26	−43
Various sources (saturated *n*-fatty acids)	12	−36	16	−35
Bacteria, usually SRB (tb fatty acids)	1	NM	1	NM
Various bacteria (diplopterol)	3	−47	4	−43

*MOX* methane-oxidizing bacteria, *SRB* sulfate-reducing bacteria, *tb* terminally-branched, *MUFAs* monounsaturated fatty acids, *av.* average, *NM* not measured

We were able to trace elongation of the MOX-specific fatty acids *n*-C_16:1ω8_ and *n*-C_16:1ω7_ to *n*-C_18_, *n*-C_20_, *n*-C_22_, *n*-C_24_, *n*-C_26_ with the same double bond positions and similar isotopic signatures in *H*. (*S*.) *methanophila*. This indicates that carbon compounds from the MOX symbionts were incorporated into their host’s biomass (Table 2, Supplementary Table S3). *I. methanophila* also showed a similar chain-elongation pattern, however, the double bond positions were not exclusively ω8 and ω7, suggesting a lower degree of carbon incorporation from its MOX symbionts than in *H*. (*S*.) *methanophila*. Given the very low isotopic values of most sponge-derived lipid biomarkers, methane appears to be the main carbon source of the Campeche sponges, and we hypothesize that the majority of the methane-derived carbon in the sponges originates from their symbionts (Supplementary Note 5).

## Conclusions

Our study revealed that two, only distantly related, species of sponges independently established highly specific, nutritional symbioses with two very closely related MOX bacteria. This convergence in symbiont acquisition underscores the strong selective advantage for these sponges in harboring MOX bacteria in the food-limited deep sea: These animals gain access to a carbon and energy source, methane, that they cannot access on their own. For the symbionts, one of the main advantages is that the sponges provide them with simultaneous and continuous access to both methane and oxygen via active water pumping and high surface to volume ratios. In contrast, free-living methanotrophs are limited to a very narrow range of habitats in which methane and oxygen co-occur.

Most research on symbioses between MOX bacteria and eukaryotes has focused on MOX from the MMG1 clade that have, so far, only been found in *Bathymodiolus* mussels. In contrast, only little is known about the symbioses between MOX from the MMG2 clade and their hosts. The diversity of hosts that these MMG2 MOX are associated with, ranging from ciliates to sponges, lobster and tubeworms, is only beginning to be recognized. Our study provides multifaceted insights into the genomic and metabolic potential of MMG2 MOX symbionts from deep-sea, seep sponges. Future studies of MMG2 MOX from other host groups will allow comparative analyses of the traits that have enabled these bacteria to independently colonize eukaryotic hosts multiple times in convergent evolution.

## Supplementary information

Supplementary File SF1

Supplementary Notes, Tables and Figures

Supplementary Table S2

Supplementary Table S3
