# Supplementary material for: Fueled by methane: deep-sea sponges from asphalt seeps gain their nutrition from methane-oxidizing symbionts
Source: ISME J. 2019 Jan 15;13(5):1209–25. doi: 10.1038/s41396-019-0346-7 (PMC6474228; doi:10.1038/s41396-019-0346-7)
Supplement: Supplementary file 1 — Supplementary File SF1 [file 41396_2019_346_MOESM1_ESM.pdf]

Supplementary File 1: Taxonomy and descriptions of the new sponge species.

Taxonomic authority restricted to Cárdenas.

These two new species have been registered under the ZooBank Life Science Identifier http://www.zoobank.org/References/0DCA7C5D-70D3-4C5D-B115-0823118E10EC.

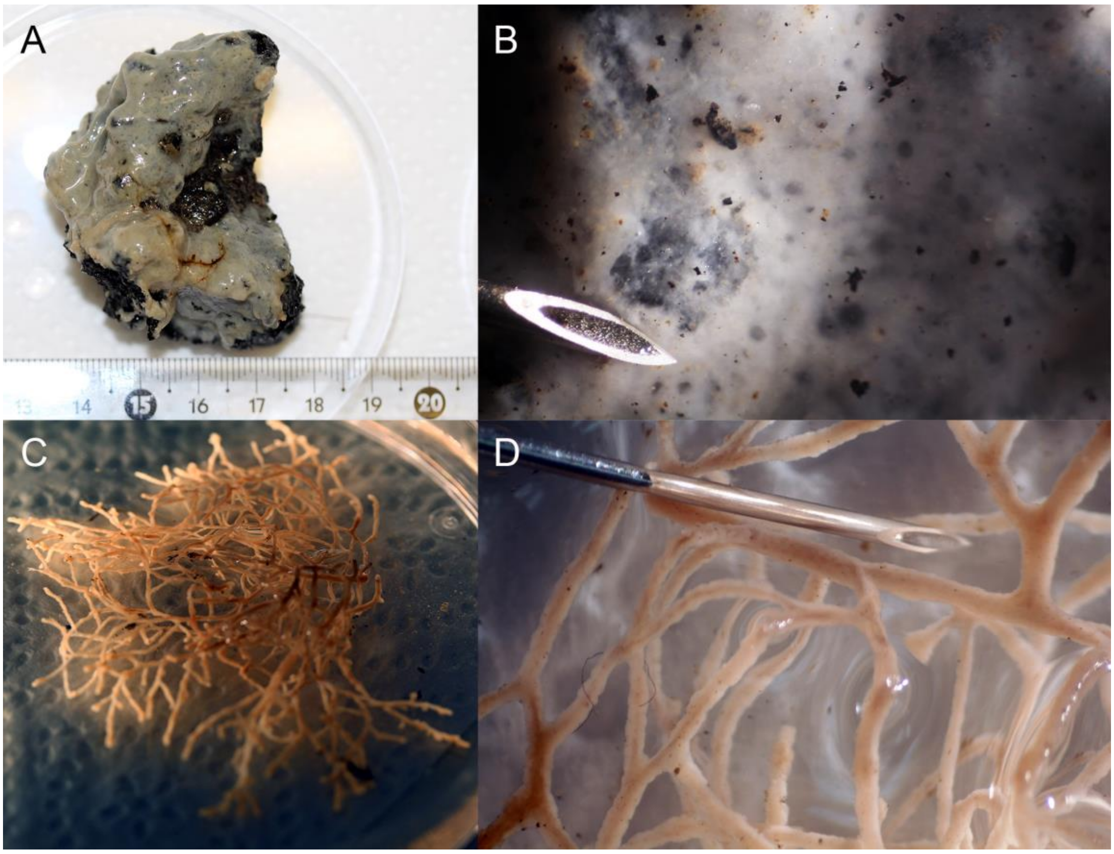

Figure SF1-1_A, One of the specimens of *Hymedesmia (Stylopus) methanophila* sp. nov. collected (not the holotype). **B,** Close-up of specimen (needle has an outer diameter of 0.6 mm). **C,** Holotype of *Iophon methanophila* sp. nov. right after collection. **D,** Close-up of holotype (needle has an outer diameter of 0.6 mm).

Phylum PORIFERA
Class DEMOSPONGIAE Sollas, 1885
Subclass HETEROSCLEROMORPHA Cárdenas, Pérez & Boury-Esnault, 2012
Order POECILOSCLERIDA Topsent, 1928

Family Hymedesmiidae Topsent, 1928
Hymedesmia Bowerbank, 1864
Subgenus *Stylopus* Fristedt, 1885

***Hymedesmia* (*Stylopus*) *methanophila* sp. nov.**
(Figures 1A-B, 2A)

TYPE MATERIAL

Holotype: (Uppsala Zoological Museum Collection) UPSZTY 167252, piece preserved in 70% ethanol and one spicule preparation. Collected in March 2015 by the ROV MARUM-Quest 4000 m, during R/V Meteor Cruise M114-2 to Campeche Knolls (21°54' N; 93°26' W), Gulf of Mexico, 2925 m depth.

OUTER MORPHOLOGY (FIG. 1A-B)

Encrusting (up to 1 mm thick) on asphalts. Color alive and in ethanol is white. Glistening surface appearance. Openings are visible on live specimens.

SPICULES (FIG. 2A)

minimum–*mean*–maximum (N=25)

Megascleres: (a) acanthostyles I, usually straight, rarely slightly bent, swollen heads with large spines (up to 3 µm long), upper half has few spines, length: 172–*194.8*–220 µm, width: 6–*7.6*–10 µm; (b) acanthostyles II, straight, head can be swollen or not, spines are regularly distributed all along the style, length: 40–*90.6*–137 µm, width: 3–*5*–7.5 µm, (c) ectodermal strongyles; one end can rarely be slightly tylote, the other rarely slightly tornote, straight, 150–*223.9*–272 µm, width in the middle: 3–*4.4*–5 µm.

No microscleres.

BARCODING

The COI and 18S rRNA gene sequences were submitted to GenBank (COI: KU659137 and 18S rRNA: MF276912) and to the Sponge Barcoding Project (http://www.palaeontologie.geo.uni- muenchen.de/SBP).

ETYMOLOGY

The name refers to the dominant sponge-associated methanotrophs.

REMARKS

Hymedesmia (Stylopus) alcoladoi van Soest, 2017 from the Guyana Shelf (64 m) is currently the only western Atlantic species of this subgenus. It is brown in ethanol (vs. white for our species) and has strict tornotes (vs. strongyles for our species). There are actually few species of this subgenus described from such depths. Two species were described from the Azores — *Hymedesmia (Stylopus) acerata* (Topsent 1904) from 880–2252 m and *Hymedesmia (Stylopus) obtusata* (Topsent, 1904) from 1360 m — and one from the coast of West Africa — *Hymedesmia (Stylopus) indivisa* (Topsent 1928) from 2165 m. *H. (S.) acerata* has tornotes, which are significantly longer (460–480 µm long) than the strongyles in our species. *H. (S.) obtusata* is black with polytylote tylotes (vs. strongyles in our species). *H. (S.) indivisa* has polytylote strongyles, which are longer (420–540 µm) than in our species.

Family Acarnidae Dendy, 1922

Iophon Gray, 1867

***Iophon methanophila* sp. nov.**
(Figures 1C-D, 2B)

TYPE MATERIAL
Holotype: (Uppsala Zoological Museum Collection) UPSZTY 167253, two spicule preparations. Collected in March 2015 by the ROV MARUM-Quest 4000 m, during R/V Meteor Cruise M114-2 to Campeche Knolls (21°54' N; 93°26' W), Gulf of Mexico, 2925 m depth.

OUTER MORPHOLOGY (FIG. 1C-D)
Branching. Color alive and in ethanol is white.

SPICULES (FIG. 2B)
minimum–*mean*–maximum (N=25)
Megascleres: (a) tylotes with elongated ends, usually with one slight bent, small swellings or spines can appear on the shaft, length: 312–*391.3*–467 µm, width: 6–*7.9*–10 µm; (b) ectodermal tylotes, straight, with flat spiny ends, 150–*180.1*–212 µm, width in the middle: 5–*5.3*–7 µm.
Microscleres: (c) palmate anisochelae, 15–*17.6*–20 µm long, width: 5–*5.7*–7.5 µm long.

BARCODING
The COI and 18S rRNA gene sequences were submitted to GenBank (COI: KU659138 and 18S rRNA: MF276914) and to the Sponge Barcoding Project.

ETYMOLOGY
The name refers to the dominant sponge-associated methanotrophs.

REMARKS
This is the only *Iophon* species without bipocillae and without styles. Megascleres are only tylotes, of two kinds.

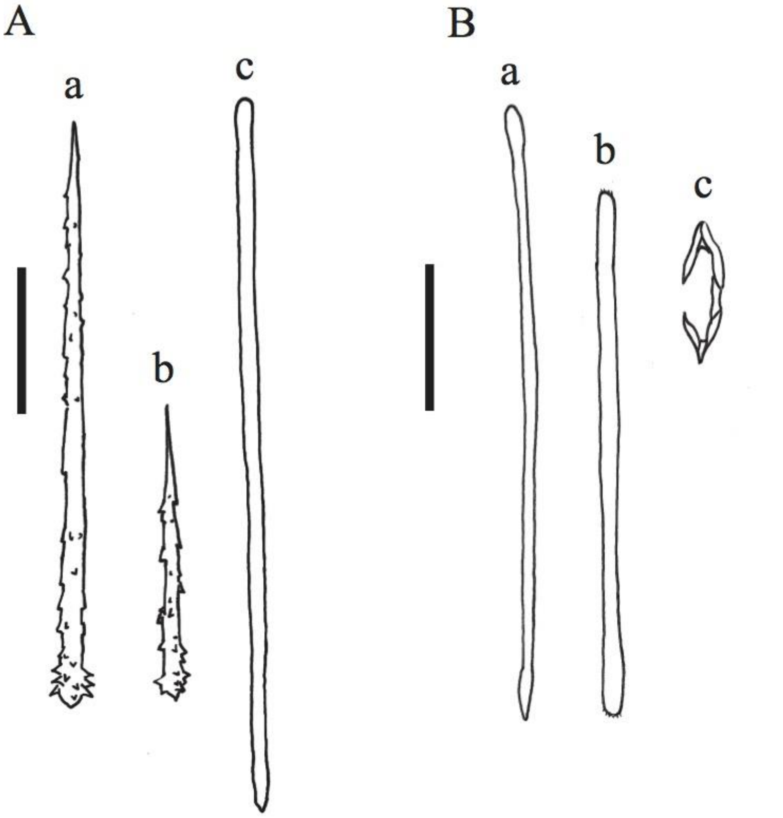

Figure SF1-2. **A,** Spicules of *Hymedesmia (Stylopus) methanophila* sp. nov. (a) acanthostyles I. (b) acanthostyles II. (c) strongyles. Scale is 50 µm for all. **B,** Spicules of *Iophon methanophila* sp. nov. (a) tylotes. (b) ectodermal tylotes. (c) anisochelae. Scale is 50 µm for (a) and (b), 25 µm for (c).

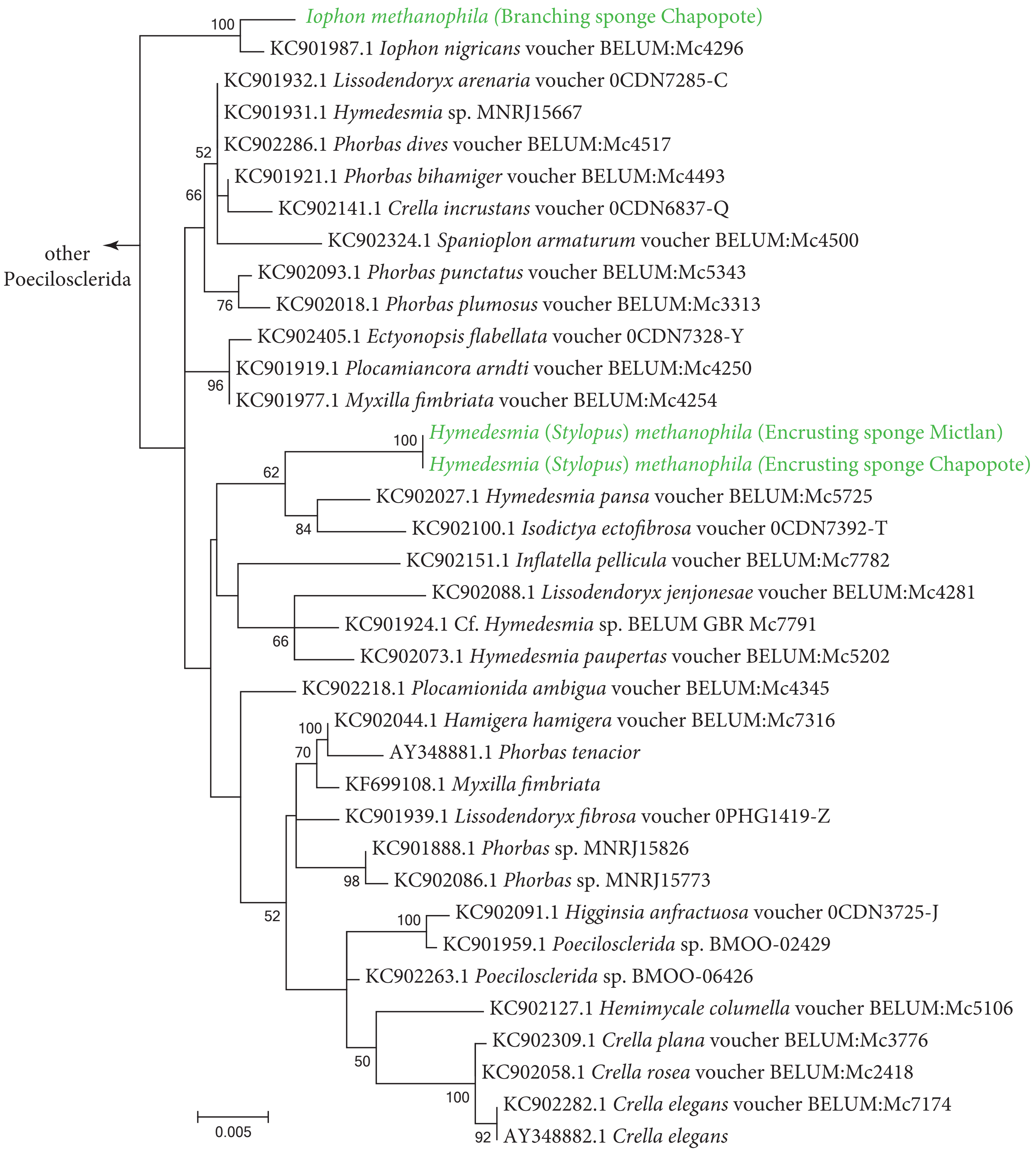

Figure SF1-3 Phylogeny of poecilosclerid sponges based on the 18S rRNA genes. The dataset included the metagenomic 18S rRNA gene sequences from this study (shown in green) and sequences from the NCBI database (103 sequences total). Bootstrap values below 50% are not shown. The tree is drawn to scale, with branch lengths representing the number of substitutions per site. The analysis included 1,553 positions.

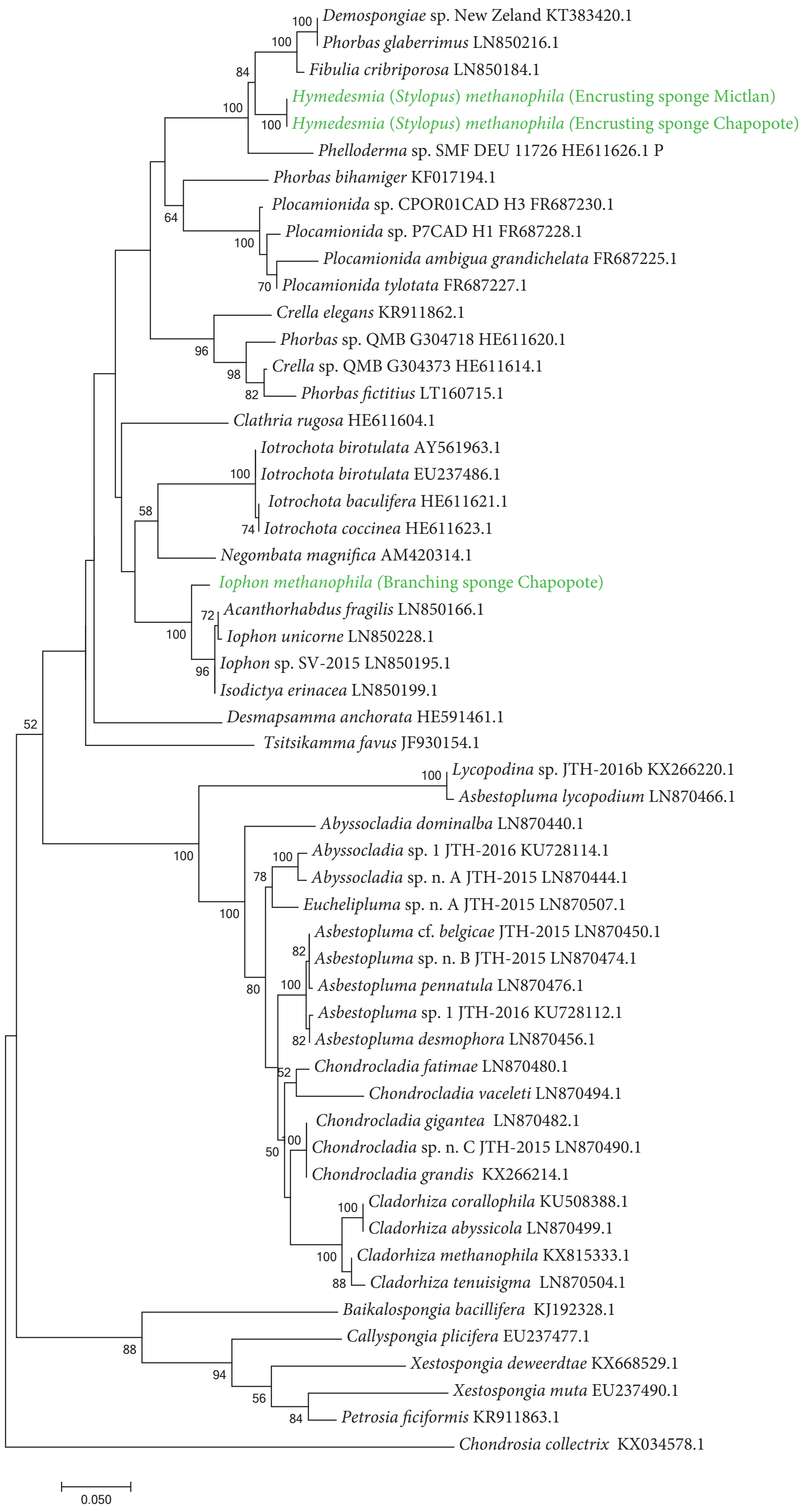

Figure SF1-4 Phylogeny of poecilosclerid sponges based on the cyctochrome c oxidase subunit I (COI) genes. The dataset included the COI gene sequences from this study (shown in green) and sequences from the NCBI database (56 sequences total). Bootstrap values below 50% are not shown. The tree is drawn to scale, with branch lengths representing the number of substitutions per site. The analysis included 466 positions.
