# Supplementary material for: Fueled by methane: deep-sea sponges from asphalt seeps gain their nutrition from methane-oxidizing symbionts
Source: ISME J. 2019 Jan 15;13(5):1209–25. doi: 10.1038/s41396-019-0346-7 (PMC6474228; doi:10.1038/s41396-019-0346-7)
Supplement: Supplementary file 2 — Supplementary Notes, Tables and Figures [file 41396_2019_346_MOESM2_ESM.pdf]

Supplementary Information for

Fueled by methane: Deep-sea sponges from asphalt seeps gain their nutrition from methane-oxidizing symbionts

Maxim Rubin-Blum[1,8*], Chakkiath Paul Antony[1], Lizbeth Sayavedra[1,9], Clara Martínez-Pérez[1], Daniel Birgel[2], Jörn Peckmann[2], Yu-Chen Wu[3], Paco Cardenas[4], Ian MacDonald[5], Yann Marcon[6], Heiko Sahling[7], Ute Hentschel[3] and Nicole Dubilier[1,7*]

[1]Max-Planck Institute for Marine Microbiology, Celsiusstrasse 1, 28359 Bremen, Germany

[2]Institute for Geology, Center for Earth System Research and Sustainability, University of Hamburg, 20146 Hamburg, Germany

[3]GEOMAR Helmholtz Centre for Ocean Research, RD3 Marine Microbiology and Christian-Albrechts University of Kiel, Düsternbrooker Weg 20, D-24105 Kiel, Germany

[4]Department of Medicinal Chemistry, Pharmacognosy, BioMedical Centre, Husargatan 3, Uppsala University, 751 23 Uppsala, Sweden

[5]Florida State University, POB 3064326, Tallahassee, FL 32306, USA

[6]Wegener Institute Helmholtz Centre for Polar and Marine Research, HGF-MPG Group for Deep Sea Ecology and Technology, Am Handelshafen 12, 27570 Bremerhaven, Germany

[7]MARUM, Center for Marine Environmental Sciences, University of Bremen, 28359 Bremen, Germany

[8]Israel Limnology and Oceanography Research, Tel Shikmona, 3108000, Haifa, Israel

[9]Quadram Institute Bioscience, Norwich Research Park, Norwich, United Kingdom

*Corresponding authors

Maxim Rubin-Blum

Phone: 0049 (0)421 2028 905
E-mail: mrubin@mpi-bremen.de

Nicole Dubilier

Phone: 0049 (0)421 2028 9032
E-mail: ndubilier@mpi-bremen.de

Contents:

1. Supplementary Notes

2. Supplementary Methods

3. Supplementary Tables

4. Supplementary Figures

Supplementary Notes

Supplementary Note 1: Sponge MOX are capable of storing methane-derived carbon in the form of glycogen: Our metabolic reconstructions, based on the genomes and transcriptomes of the sponge MOX symbionts, suggest that the intracellular vacuoles we observed in the symbionts with transmission electron microscopy contained glycogen. The genes needed to synthesize glycogen were present and expressed in the sponge MOX symbionts (*glgC*, *glgA* and *glgB* genes, which encode the glucose-1-phosphate adenylyltransferase, glycogen synthase and 1,4-alpha-glucan branching enzyme, respectively). In addition to glycogen, many bacteria also store carbon in the form of polyhydroxyalkanoates (PHAs). However, type I methanotrophs have so far not been shown to be able to synthesize PHAs [1]. Correspondingly, no genes that encode PHA synthases were found in the genomes of the sponge-associated MOX.

Supplementary Note 2: Convergence of respiratory nitrate reductases in the sponge and mussel symbionts: Genome comparisons of symbiotic and free-living MOX revealed that a *NarGHIJ* gene cluster that encodes a respiratory nitrate reductase (Nar) was ubiquitously found in the genomes of MOX hosted by both sponges and mussels, while rarely present in those of free-living gammaproteobacterial MOX (7 out of 54 publicly available genomes). Comparative phylogenetic analyses of the *narG* gene revealed that the sequences from sponge and mussel MOX symbionts formed a monophyletic clade with *Methyloprofundis sedimentii*, a MOX isolated from whale falls that is very closely related to the mussel MOX symbionts [2]. In contrast, the *narG* gene sequences from all other free-living MOX whose genomes have been sequenced belong to a very distant clade (**Supplementary Figure S9**). Phylogenetic clustering of the narG genes in mussel and sponge MOX symbionts suggests a high level of functional convergence between their Nar protein complexes and indicates adaptation via kinetic specialization to different substrate levels within hosts than in the free-living environment. Similar to Nar from pathogenic bacteria of humans, these proteins may allow the symbiotic MOX to survive hypoxia and mitigate exposure to reactive nitrogen species [3].

Supplementary Note 3: Genes unique to the sponge-associated MOX: The majority of orthologous genes unique to the MOX symbionts from both sponge species were poorly-annotated short ORFs with a median length of 240 bp. These ORFs were not found in free-living relatives, e.g. two MMG2 MOX from the North Sea sandy sediments enrichments, as well as *Methylomarinum*, *Methylomonas*, *Methylobacter* and *Methylomicrobium* spp.. Some of these ORFs were homologous to phage genes and phage defense systems, such as clustered regularly interspaced short palindromic repeats (CRISPRs) and sequences that encoded CRISPR-associated proteins Cas, as well as restriction-modification systems. Other unique genes were involved in

exopolysaccharide production (e.g. glycosyltransferases, oligosaccharyltransferase , capsule polysaccharide export proteins etc). These highly variable capsule polysaccharides, such as O-antigens, are suggested to play a role in interactions between pathogenic and symbiotic bacteria and their hosts [4, 5]. Alternatively, the variable capsular polysaccharides are also known to play a role in the interaction between bacteria and their phages [6]. Thus, many of the annotatable genes unique to the sponge MOX symbionts may be involved in interactions with their animal hosts or with phages.

Supplementary Note 4: Fatty acids and sterols in Campeche sponges: The most abundant fatty acids in *H.* (*S.*) *methanophila* were the short-chain *n*-fatty acids $C_{16:1\omega8}$ and $C_{16:1\omega7}$, as well as the long-chain *n*-$C_{26:3\Delta5,9,?}$ fatty acid. The two fatty acids *n*-$C_{16:1\omega8}$ and *n*-$C_{16:1\omega7}$ were accompanied by a series of ω8 and ω7 *n*-fatty acids, ranging from C_{18} to C_{26}. The most prominent Δ5,9 fatty acid *n*-$C_{26:3\Delta5,9,?}$ was accompanied by other Δ5,9 fatty acids and two brominated Δ5,9 fatty acids (**Supplementary Figure S10**). The fatty acid pattern of *I. methanophila* was somewhat different, with *n*-$C_{28:3\Delta5,9,?}$ as the most prominent fatty acid, accompanied by *n*-$C_{16:1\omega8}$ and saturated *n*-C_{16} fatty acids. *I. methanophila* also contained various *n*-fatty acids with 18 carbon atoms and one and two double bonds (**Supplementary Figure S10**). A series of monounsaturated *n*-fatty acids from C_{20} to C_{24} also occurred in the *I. methanophila*, and the double bond positions of the various fatty acids were more variable than in *H.* (*S.*) *methanophila.*

The sterols of *H.* (*S.*) *methanophila* comprised compounds with double bond positions at carbons 5 and 24 (Δ5,24 sterols), with 24-methyl-cholesta-5,24-*dien*-3β-ol as major sterol, accompanied by the minor Δ5,24 sterols desmosterol, fucosterol and *iso*-fucosterol, as well as cholesterol (see **Supplementary Table S4**). The C_{30}-hopanoid diplopterol was the only cyclic non-sterol compound detected. *H.* (*S.*) *methanophila* also contained 24-methyl-cholesta-5,24-*dien*-3β-ol as a major sterol, which was accompanied by only cholesterol and diplopterol. Since diplopterol was identified in both sponges, both samples were analyzed for bacteriohopanepolyols with coupled high-performance liquid chromatography mass spectrometry, but no bacteriohopanepolyols were identified.

Supplementary Note 5: Stable isotope composition of bulk tissue: $\delta^{13}C$ and $\delta^{15}N$ values of sponge bulk tissue were −40.6‰ and 1.7‰ for the *H.* (*S.*) *methanophila* individual from Chapopote, −37.1‰ and 2.7‰ for the *H.* (*S.*) *methanophila* individual from Mictlan and −42.0‰ and 0.0‰ for the *I. methanophila* individual from Chapopote. The $\delta^{13}C$ value of asphalt encrusted by sponges was −27.6‰ (nitrogen was below detection limits). Bulk tissues of encrusting sponges contained asphalt impurities, which may have introduced errors to the bulk $\delta^{13}C$ measurement, resulting in $\delta^{13}C$ values that were more positive than those of the corresponding fatty acids. For comparison, bulk tissue $\delta^{13}C$ and $\delta^{15}N$ values of *B. heckerae*, which co-occur with sponges at Chapopote

averaged −47.0±5.0‰ and 1.5±1.2‰. Given that bathymodiolin mussels acquire their carbon as well as nitrogen from their symbionts [7], the very similar $\delta^{15}N$ values between Campeche mussels and sponges indicate that the latter most likely also gain their nutrition from their symbionts.

Supplementary Note 6: Campeche sponges gain their nutrition from the symbiotic MOX, rather than filtering free-living MOX: The isotopic signatures of Campeche sponges, as well as the elongation patterns of their fatty acids suggest that their MOX symbionts contribute significantly to their nutrition. While we cannot entirely exclude filter-feeding on free-living MOX as an additional source of nutrition, several lines of evidence suggest that the symbionts provided their hosts with most of their nutrition. First, we found very few Illumina reads that mapped to the 16S rRNA gene sequences of MOX bacteria other than the symbiotic ones. These reads represented only a small fraction of all the low-abundance 16S rRNA reads from free-living microbes that had been likely retained by the sponges. Second, if the sponges gained a significant amount of their nutrition from filter-feeding, their carbon isotopic signatures should not have been in the same range as that of methane, but rather heavier, given the non-selective filtration behavior of sponges, and the diversity of autotrophic and heterotrophic microorganisms in seep waters. A third line of evidence for the nutritional dependence of the sponges on their symbiotic MOX is the strong dilution of external MOX signal at the sterol composition level. Many Type I methanotrophs, including the symbionts of bathymodiolin mussels, are able to synthesize 4-methylated sterols [8, 9]. The sponge-associated MOX appeared to lack the key enzymes that catalyze the synthesis of 4-methylated sterols (**Supplementary Figure S11**). We were not able to detect 4-methylated sterols in the lipid extracts from Campeche sponges, confirming our metabolic reconstruction. Given that it is unlikely that the free-living MOX in the vicinity of the sponges were not capable of 4-methylated sterol synthesis, the external MOX sterol signal was strongly diluted by that of the symbiotic MOX, confirming the very low contribution of non-symbiotic MOX to the sponge biomass. Fourth, our ultrastructural observations revealed that the MOX morphotypes in the sponges were in various stages of digestion. These digestion stages were often observed when the symbiotic MOX morphotypes were present in high densities in the mesohyl matrix, suggesting direct feeding on the symbiotic MOX.

Supplementary Methods

Supplementary Methods 1, downstream genome analyses: The genomes were annotated with RAST [10] and the DOE-JGI Microbial Genome Annotation Pipeline [11]. Annotations were manually cross-checked and verified using NCBI's BLAST [12]. Average nucleotide identity (ANI) values were calculated with the ANI calculator (http://enve-omics.ce.gatech.edu/ani/). Conserved domains were annotated with NCBI CD-search [13]. Pangenomes were analyzed with panX [14]. Toxin-antitoxin systems were identified with TAfinder using default parameters

(http://202.120.12.133/TAfinder/index.php). Polymorphic toxin systems were identified as described previously [15]. Clusters of orthologous groups (COGs) annotation for each individual genome was performed with eggNOG-mapper [16, 17]. COG counts were normalized to their sum in each genome, and principal component analysis was performed with PAST3 [18].

The relative 16S rRNA read abundances in the metagenomes and metatranscriptomes were determined as follows: Illumina reads were mapped to the SILVA132 small subunit (SSU) rRNA gene database [19] with phyloFlash (https://github.com/HRGV/phyloFlash) using 0.7 identity cutoff. The mapped reads were assembled with Spades V3.10 [20, 21]. Metagenomic and metetranscriptomic reads were re-mapped to the assembled 16S rRNA gene sequences using BBMap (Bushnell B, http://sourceforge.net/projects/bbmap/) with 0.98 minimum identity, and coverage values for each individual sequence were normalized to their sum. To validate if we have assembled the16S rRNA gene sequence for the most of phyla with the assembly-independent method implemented in phyloFlash, that is, estimating the number of reads that mapped to each sequence in the SILVA132 database (the relative abundance of mapping-based taxa. To validate if the16S rRNA gene sequences were assembled for the most of the phyla: 1) We verified if each metagenomics bin contained a 16S rRNA gene and 2) We compared the phylogenetic diversity revealed by the assembly of the 16S rRNA gene sequences with that determined by the assembly-independent relative abundance of mapping-based taxa (that is, the number of reads that mapped to each sequence in the SILVA132 database). To estimate abundance by genome coverage, we mapped metagenomic reads using BBmap with 0.98 minimum identity to each metagenome assembled genome (MAG), and divided by the total number of reads that mapped to all the MAGs assembled for each library.

Supplementary Methods 2, phylogenies: Maximum Likelihood phylogenies were reconstructed with MEGA7 [22], based on the best-predicted model, following alignment of metagenomics sequences and representative sequences in the NCBI database by MAFFT [23]. The percentage of trees in which the associated taxa clustered together was determined based on 1,000 bootstrap resamplings.

Supplementary Methods 3, bulk tissue stable isotope, and elemental C and N measurements: Samples were acidified overnight over fuming HCL in a desiccator, oven-dried overnight at 50°C, and transferred to tin cups for analysis. Organic carbon and nitrogen content and bulk carbon and nitrogen isotopic compositions ($\delta^{13}C$ and $\delta^{15}N$) were measured using a carbon-hydrogen-nitrogen (CHN) elemental analyzer (Thermo Flash EA 1112) coupled with a continuous flow isotope ratio mass spectrometer (Thermo Delta Plus XP, Thermo Fisher Scientific, Schwerte, Germany). USG40

and caffeine standards, calibrated against international standards (IAEA N1 and N2), were measured every 4-6 samples to ensure the accuracy of measured values (~ 10 caffeine standards and ~5 USGS 40 international standards per mass spec run).. Instrument precision was 0,15 ‰ for $\delta^{13}C$ and 0,08‰ for $\delta^{15}N$, based on replicate analyses of standards measured in parallel with the samples. As a quality control, measurement runs where the standard deviation of the standards exceeded 0.2 ‰ were not analyzed.

Supplementary Methods 4, Chapopote mosaic mapping

Photographs used for the photomosaic were acquired with the ROV MARUM-Quest during cruise M114/2 (Station 90-1, GeoB: 19333-1) using a downward-facing PROSILICA GT6600 with 29 megapixels (6576 x 4384) resolution. The photographs were taken from an altitude of 2.5m above the seafloor. The photomosaic was constructed with the LAPM Tool [24, 25] from 133 photographs. The mosaic was georeferenced using Ultra Short Baseline (USBL) as well as Doppler Velocity Log (DVL) acoustic positioning.

References

1. Pieja AJ, Rostowski KH, Criddle CS. Distribution and selection of poly-3-hydroxybutyrate production capacity in methanotrophic proteobacteria. *Environ Microbiol* 2011; **62**: 564–573.

2. Tavormina PL, Hatzenpichler R, McGlynn S, Chadwick G, Dawson KS, Connon SA, et al. *Methyloprofundus sedimenti* gen. nov., sp. nov., an obligate methanotroph from ocean sediment belonging to the 'deep sea-1' clade of marine methanotrophs. *Int J Syst Evol Microbiol* 2015; **65**: 251–259.

3. Vázquez-Torres A, Bäumler AJ. Nitrate, nitrite and nitric oxide reductases: from the last universal common ancestor to modern bacterial pathogens. *Curr Opin Microbiol* 2016; **29**: 1–8.

4. Kalynych S, Morona R, Cygler M. Progress in understanding the assembly process of bacterial O-antigen. *FEMS Microbiol Rev* 2014; **38**: 1048–1065.

5. Lerouge I, Vanderleyden J. O-antigen structural variation : mechanisms and possible roles in animal / plant-microbe interactions. *FEMS Microbiol Rev* 2001; **26**: 17–47.

6. Rodriguez-Valera F, Martin-Cuadrado A-B, Rodriguez-Brito B, Pasić L, Thingstad TF, Rohwer F, et al. Explaining microbial population genomics through phage predation. *Nat Rev Microbiol* 2009; **7**: 828–836.

7. Becker EL, Lee RW, Macko SA., Faure BM, Fisher CR. Stable carbon and nitrogen isotope compositions of hydrocarbon-seep bivalves on the Gulf of Mexico lower continental slope. *Deep Sea Res Part II Top Stud Oceanogr* 2010; **57**: 1957–1964.

8. Kellermann MY, Schubotz F, Elvert M, Lipp JS, Birgel D, Prieto-Mollar X, et al. Symbiont–host relationships in chemosynthetic mussels: A comprehensive lipid biomarker study. *Org Geochem* 2012; **43**: 112–124.

9. Takishita K, Takaki Y, Chikaraishi Y, Ikuta T, Ozawa G, Yoshida T, et al. Genomic evidence that methanotrophic endosymbionts likely provide deep-sea *Bathymodiolus* mussels with a sterol intermediate in cholesterol biosynthesis. *Genome Biol Evol* 2017; **9**: 1148–1160.

10. Overbeek R, Olson R, Pusch GD, Olsen GJ, Davis JJ, Disz T, et al. The SEED and the Rapid Annotation of microbial genomes using Subsystems Technology (RAST). *Nucleic Acids Res* 2014; **42**: D206–D214.

11. Huntemann M, Ivanova NN, Mavromatis K, Tripp HJ, Paez-Espino D, Palaniappan K, et al. The standard operating procedure of the DOE-JGI Microbial Genome Annotation Pipeline (MGAP v.4). *Stand Genomic Sci* 2015; **10**: 86.

12. Johnson M, Zaretskaya I, Raytselis Y, Merezhuk Y, McGinnis S, Madden TL. NCBI BLAST: a better web interface. *Nucleic Acids Res* 2008; **36**: W5–W9.

13. Marchler-Bauer A, Bryant SH. CD-Search: Protein domain annotations on the fly. *Nucleic Acids Res* 2004; **32**: 327–331.

14. Ding W, Baumdicker F, Neher RA. panX: pan-genome analysis and exploration. *Nucleic Acid Res,* 2018; **46**; e5.

15. Sayavedra L, Kleiner M, Ponnudurai R, Wetzel S, Pelletier E, Barbe V, et al. Abundant toxin-related genes in the genomes of beneficial symbionts from deep-sea hydrothermal vent mussels. *Elife* 2015; **4**.

16. Huerta-Cepas J, Forslund K, Coelho LP, Szklarczyk D, Jensen LJ, Von Mering C, et al. Fast genome-wide functional annotation through orthology assignment by eggNOG-mapper. *Mol Biol Evol* 2017; **34**: 2115–2122.

17. Huerta-Cepas J, Szklarczyk D, Forslund K, Cook H, Heller D, Walter MC, et al. EGGNOG 4.5: A hierarchical orthology framework with improved functional annotations for eukaryotic, prokaryotic and viral sequences. *Nucleic Acids Res* 2016; **44**: D286–D293.

18. Hammer Ø, Harper DAT, Ryan PD. PAST: Paleontological Statistics Software Package for Education and Data Analysis. *Palaeontol Electron* 2001; **4(1)**: 1–9.

19. Glöckner FO, Yilmaz P, Quast C, Gerken J, Beccati A, Ciuprina A, et al. 25 years of serving the community with ribosomal RNA gene reference databases and tools. *J Biotechnol* 2017; **261**: 169–176.

20. Nurk S, Bankevich A, Antipov D. Assembling genomes and mini-metagenomes from highly chimeric reads. *Res Comput Mol Biol* 2013; 158–170.

21. Bankevich A, Nurk S, Antipov D, Gurevich AA., Dvorkin M, Kulikov AS, et al. SPAdes: A new genome assembly algorithm and its applications to single-cell sequencing. *J Comput Biol* 2012; **19**: 455–477.

22. Kumar S, Stecher G, Tamura K. MEGA7: Molecular Evolutionary Genetics Analysis version 7.0 for bigger datasets. *Mol Biol Evol* 2016; **33**: msw054.

23. Katoh K, Standley DM. MAFFT multiple sequence alignment software version 7: Improvements in performance and usability. *Mol Biol Evol* 2013; **30**: 772–780.

24. Marcon Y, Sahling H, Bohrmann G. Model Development LAPM: a tool for underwater large-area photo-mosaicking. *Geosci Instrumentation, Methods Data Syst* 2013; **2**: 189–198.

25. Marcon Y. LAPMv2: An improved tool for underwater large-area photo-mosaicking. *Proc. MTS/IEEE Ocean. 2014 Conf. St. John's, Newfoundland, Canada.* 2014.

Supplementary Tables

Supplementary Table S1 Genome assembly statistics. GC, guanine + cytosine; bp, base pairs; mbp, million base pairs.

**Host**	*Million DNA reads*	*Million RNA reads*	*# contigs (≥1000 bp)*	*Total length (≥ 1000 bp)*	*Largest contig (mbp)*	*GC (%)*	*N50 (mbp)*	*Completeness (%)*	*Contamination (%)*
Encrusting sponge Chapopote	28	30	63	2.22	0.22	37.76	0.05	96.5	0.3
Encrusting sponge Mictlan	29	38	82	2.01	0.11	37.83	0.04	95.0	0.3
Branching sponge Chapopote 1	43	n.a.	126	2.02	0.12	37.68	0.04	94.6	0.5
Branching sponge Chapopote 2	41	n.a.	116	2.09	0.12	37.73	0.05	95.6	0.5

Supplementary Table S2 (Excel): Eukaryote-like domains in sponge MOX genomes and their expression values. ORF, open reading frame; TPM, transcripts per million.

Supplementary Table S3 (Excel): Unique genes in core genomes of the free-living ('Methylomonadaceae' + Marine Methylotrophic Group II sediment enrichment genomes) and the sponge-associated methane oxidizers.

Supplementary Table S4 Composition of lipid biomarkers and their $\delta^{13}C$ values in sponge tissue. MOX, methane-oxidizing bacteria; ND, not detected; NM, not measured; *, co-elution of various monounsaturated fatty acids leads to a mixture of $\delta^{13}C$ values of neighboring fatty acids; i, *iso*-, bm, biomass.

		H. (S.) methanophila		*I. methanophila*	
Compounds	Putative source(s)	Contents (µg/g bm)	$\delta^{13}C$ (‰)	Contents (µg/g bm)	$\delta^{13}C$ (‰)
saturated fatty acids					
n-C_{14}	various	0.11	-39	0.19	-36
n-C_{15}	various	0.10	-43	0.12	NM
n-C_{16}	various	0.38	-36	1.99	-35
n-C_{17}	various	ND		0.13	-35
n-C_{18}	various	0.18	-28	0.67	-34
n-C_{20}	various	ND		0.20	-35
n-C_{21}	various	ND		0.07	NM
n-C_{22}	various	ND		0.35	-38
n-C_{23}	various	ND		0.15	NM
n-C_{24}	various	ND		0.24	-36
n-C_{25}	various	ND		0.13	NM
n-C_{26}	various	ND		0.27	NM

n-C_{28}	various	ND		0.40	NM
terminally-branched fatty acids					
iso-C_{15}	bacteria	0.02	NM	0.05	NM
anteiso-C_{15}	bacteria	0.03	NM	0.07	NM
iso-C_{17}	bacteria	ND		0.05	NM
anteiso-C_{17}	bacteria	ND		0.07	NM
monounsaturated fatty acids					
n-$C_{16:1\omega8}$	MOX	1.03	-46*	1.86	-51
n-$C_{16:1\omega7}$	MOX	0.69	-46*	0.26	-48
n-$C_{16:1\omega6}$	MOX	0.20	-47*	0.07	-50
n-$C_{16:1\omega5}$	MOX	0.12	-47*	0.09	-49
n-$C_{18:1\omega9}$	sponge	ND		0.31	-34
n-$C_{18:1\omega8}$	MOX	0.22	-44*	ND	
n-$C_{18:1\omega7}$	MOX	0.27	-44*	0.68	-49
n-$C_{20:1\omega8}$	sponge	0.03	-47*	0.16	-46*
n-$C_{20:1\omega7}$	sponge	0.06	-47*	0.38	-46*
n-$C_{20:1\omega6}$	sponge	ND		0.11	-46*
n-$C_{22:1\omega9}$	sponge	0.08		0.24	-36
n-$C_{22:1\omega8}$	sponge	0.08	-47*	ND	
n-$C_{22:1\omega7}$	sponge	0.20	-47*	0.31	-40
n-$C_{24:1\omega9}$	sponge	ND		0.34	-51
n-$C_{24:1\omega8}$	sponge	0.14	-48*	ND	
n-$C_{24:1\omega7}$	sponge	0.30	-48*	0.25	-51
n-$C_{26:1\omega8}$	sponge	0.05	-47*	ND	
n-$C_{26:1\omega7}$	sponge	0.04	-47*	ND	
demospongic acids					
n-$C_{16:2\Delta5,9}$	sponge	0.18	-46	0.79	-42
n-$C_{18:2\Delta5,9}$	sponge	ND		0.59	-51
n-$C_{20:2\Delta?}$	sponge	ND		0.26	-44
n-$C_{26:3\Delta5,9,?\ (18)}$	sponge	0.90	-47	ND	
n-$C_{26:3\Delta5,9,19}$	sponge	0.00	-47*	ND	
n-$C_{26:2\Delta5,9}$	sponge	0.13	-47*	ND	

br-$C_{26:3\Delta 5,9,19}$	sponge	0.21	-47	ND	
br-$C_{26:2\Delta 5,9}$	sponge	0.08	-46	ND	
n-$C_{28:3\Delta 5,9,?}$ (21, 19)	sponge	ND		2.89	-51
Sterols					
cholesterol	sponge	0.15	-40	2.10	-43
cholesta-5,24-*dien*-3β-ol (desmosterol)	sponge	0.10	-43	ND	
24-methyl-cholesta-5,24-*dien*-3β-ol	sponge	1.84	-40	3.30	-43
24-ethylcholesta-5,24*E*-*dien*-3β-ol (fucosterol)	sponge	0.32	-42	ND	
24-ethylcholesta-5,24*Z*-*dien*-3β-ol (*i*-fucosterol)	sponge	0.05	-42	ND	
Hopanols					
Diplopterol	bacteria	0.26	-47	0.90	-43

Supplementary Figures

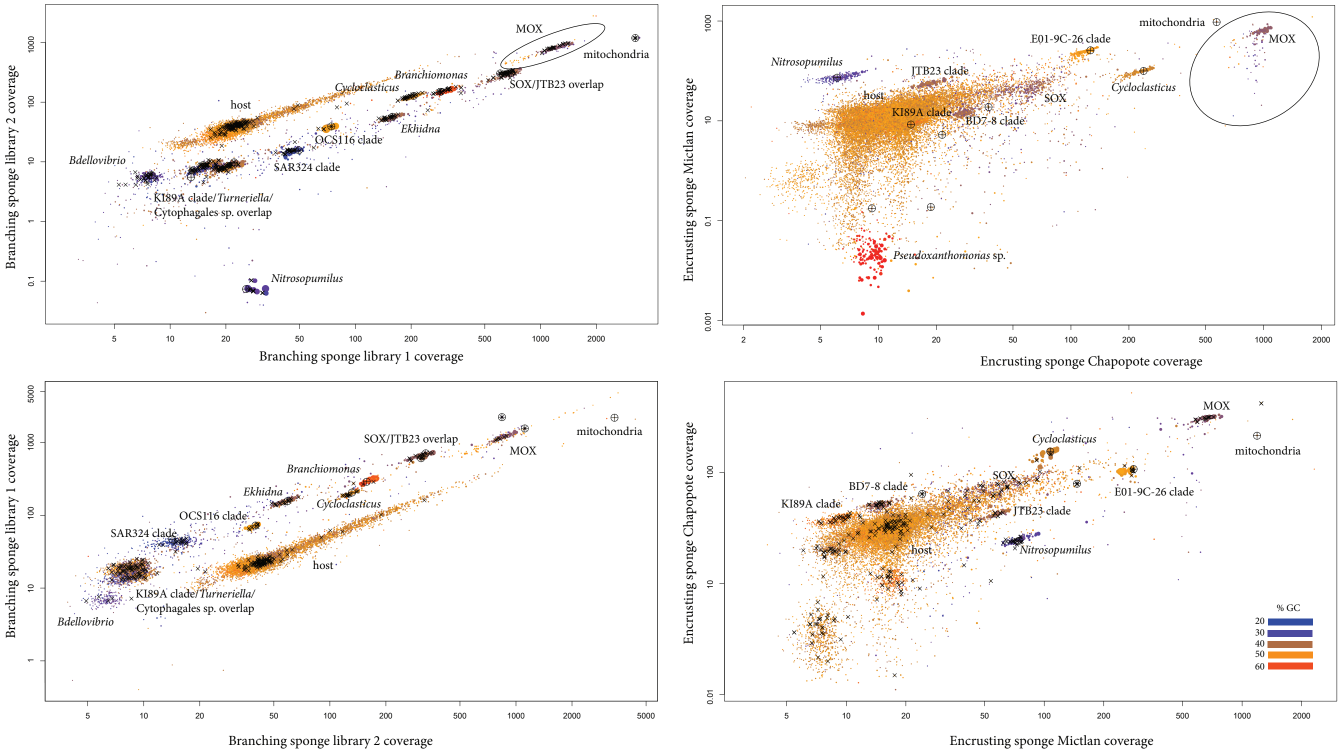

Supplementary Figure S1 Differential sequencing coverage of > 2000 bp scaffolds from the sponge metagenomic libraries. Each circle represents a metagenomic scaffold. The circles are colored according to their GC content, the diameter of these circles is proportional to scaffold length. ‘x’ symbols represent scaffolds that contain tRNAs, crossed circles represent scaffolds that contain 16S rRNA genes. Ellipses show examples of bins chosen for reassembly of sponge MOX symbionts. The sizes of the complete bins shown here (all contigs included in the ellipse) are 2.44 Mbp for the branching sponge and 2.29 Mbp for the encrusting sponge.

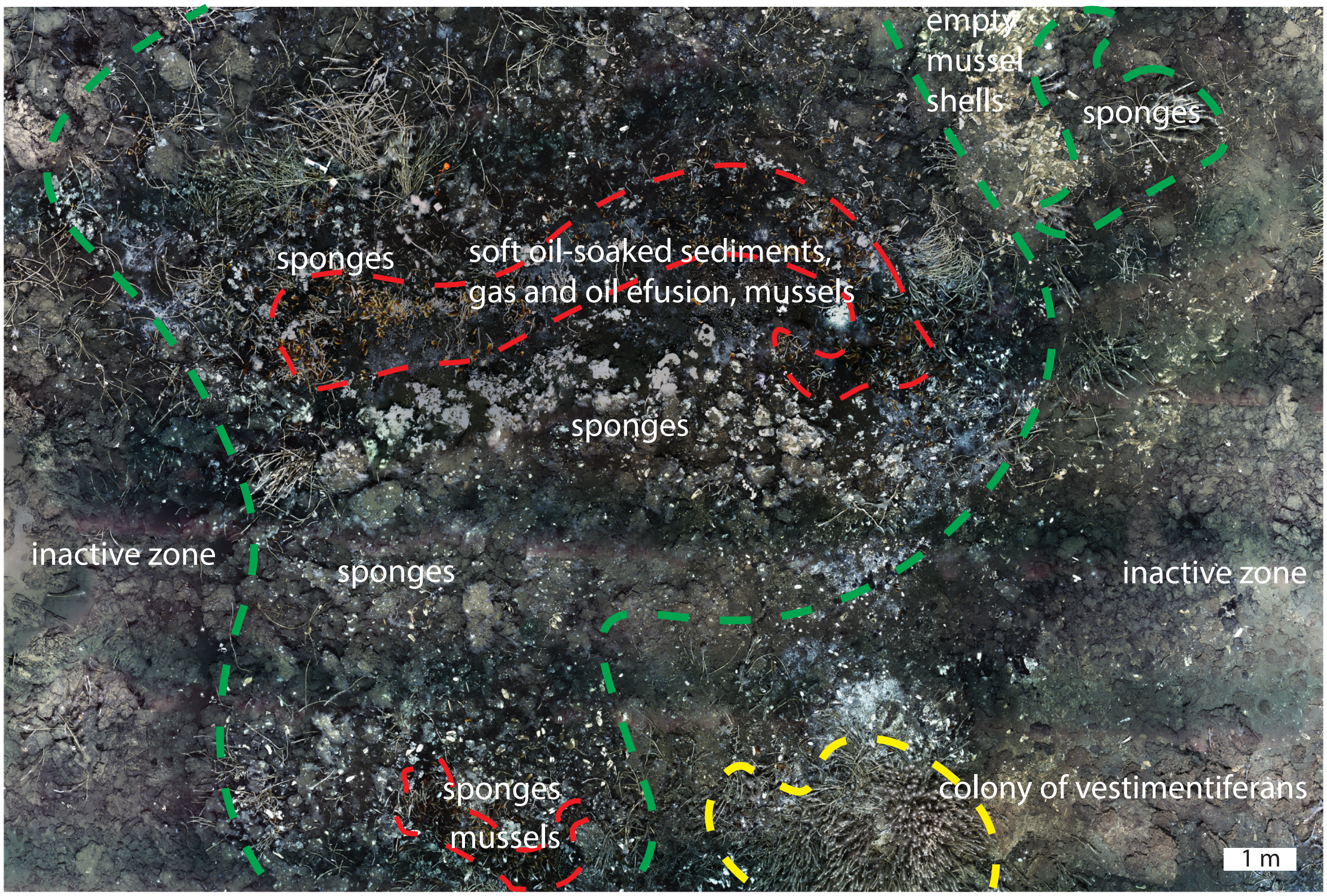

Supplementary Figure S2 A photomosaic of the Chapopote 'bubble' site where the sponges were collected (photos taken from 2.5 m above the seafloor). Areas dominated by sponges (green), mussels (red) and tubeworms (yellow) are highlighted.

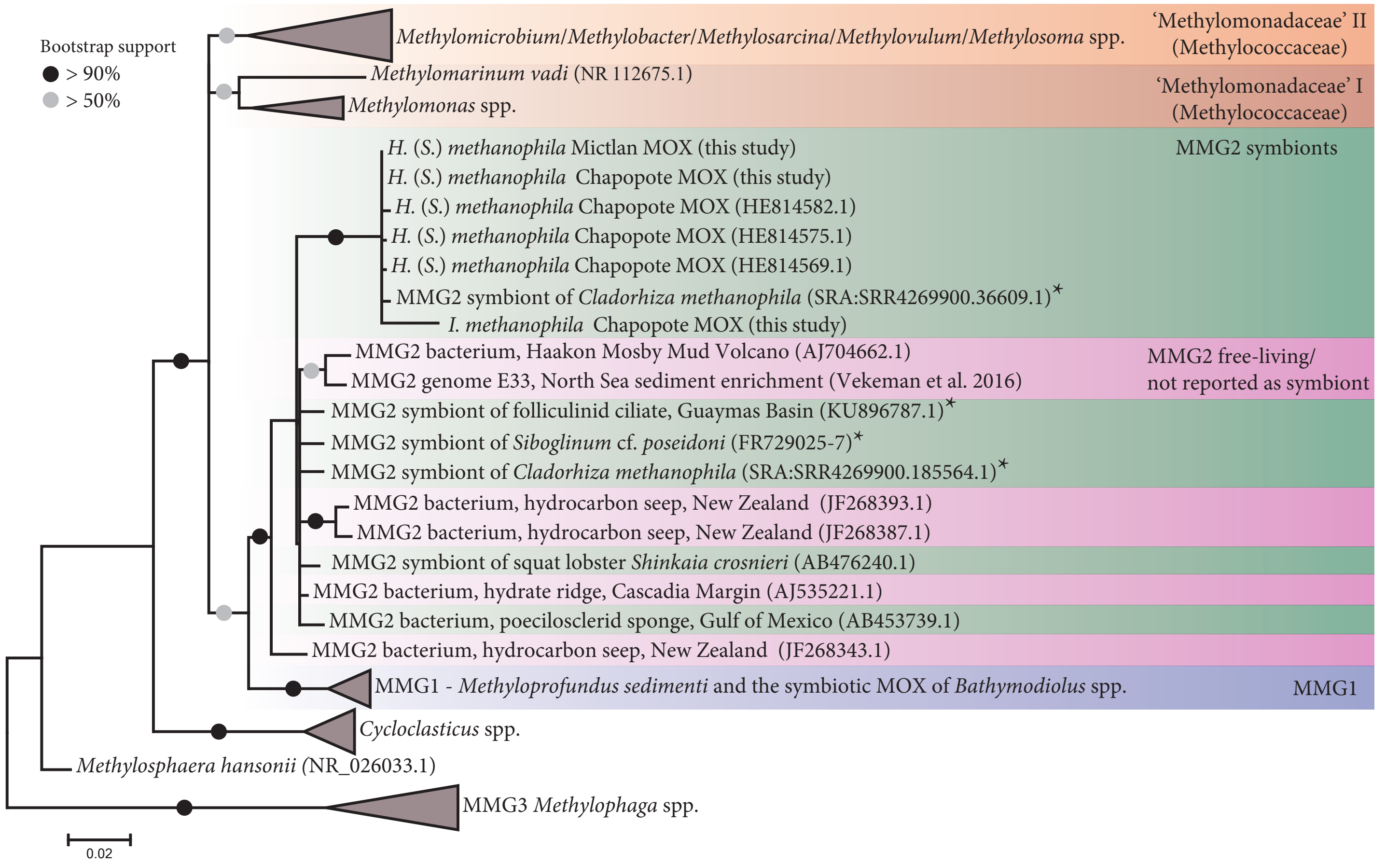

Supplementary Figure S3 Phylogeny of the 16S rRNA genes from the symbiotic MOX of sponges collected at the Campeche and Mictlan seeps. The dataset included metagenomic 16S rRNA gene sequences from this study and sequences from the NCBI database (94 sequences total). Bootstrap values below 50% are not shown. The tree is drawn to scale, with branch lengths representing the number of substitutions per site. The analysis included 1,262 positions. The genome of the North Sea sediment enrichment genome D14 lacked the fully assembled 16S rRNA gene sequence. Star marks the 16S rRNA gene sequences which were excluded from the tree calculation due to its short length, which would have hindered the phylogeny resolution, and whose position in the tree was estimated based on a phylogeny of separately aligned truncated sequences. MMG is Marine Methylotrophic Group.

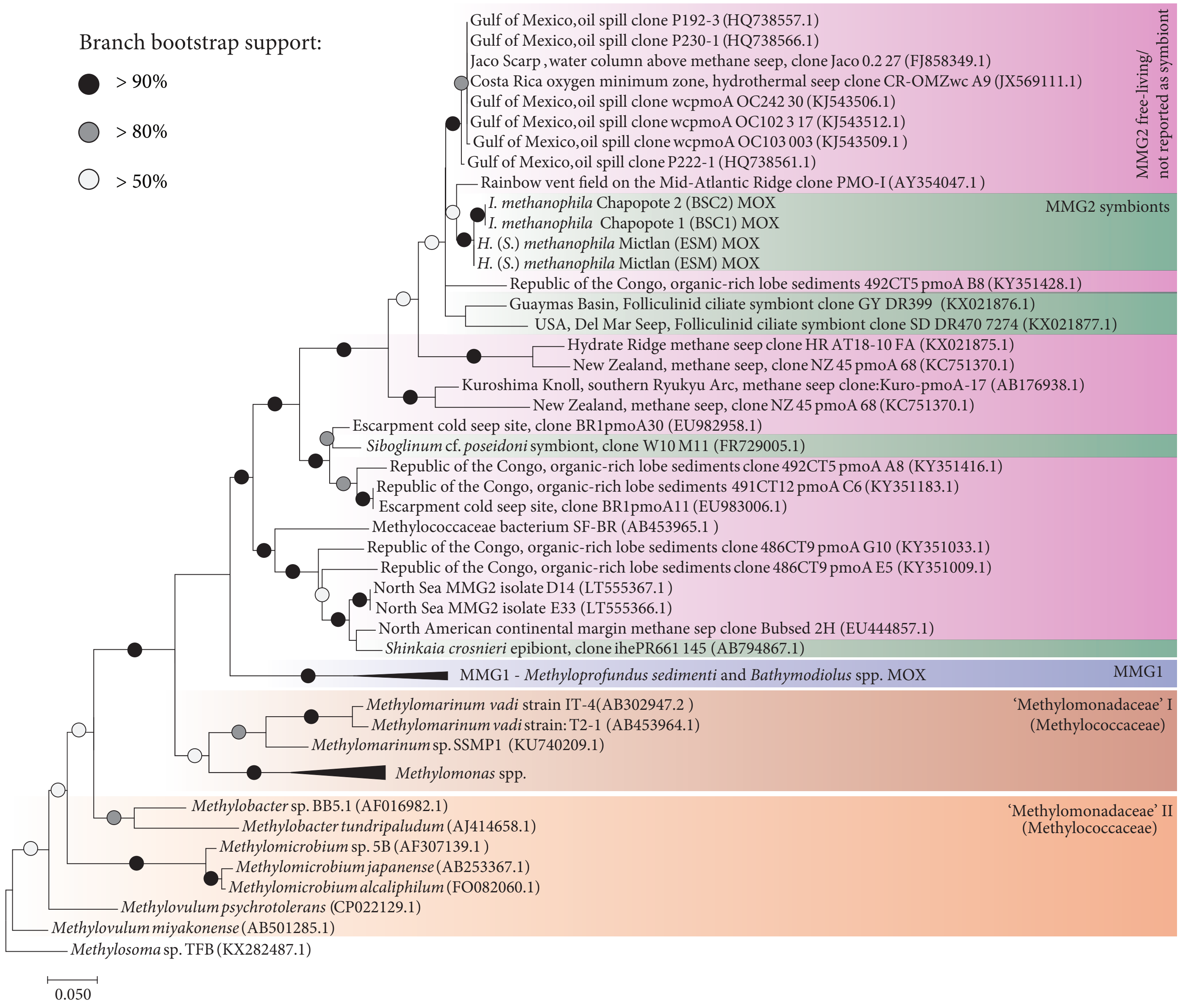

Supplementary Figure S4a Phylogeny of *pmoA* genes from the symbiotic MOX of sponges collected at the Campeche and Mictlan seeps. The dataset included *pmoA* sequences from this study and from the NCBI database (54 nucleotide sequences total). The tree is drawn to scale, with branch lengths representing the number of substitutions per site. The analysis included 344 positions.

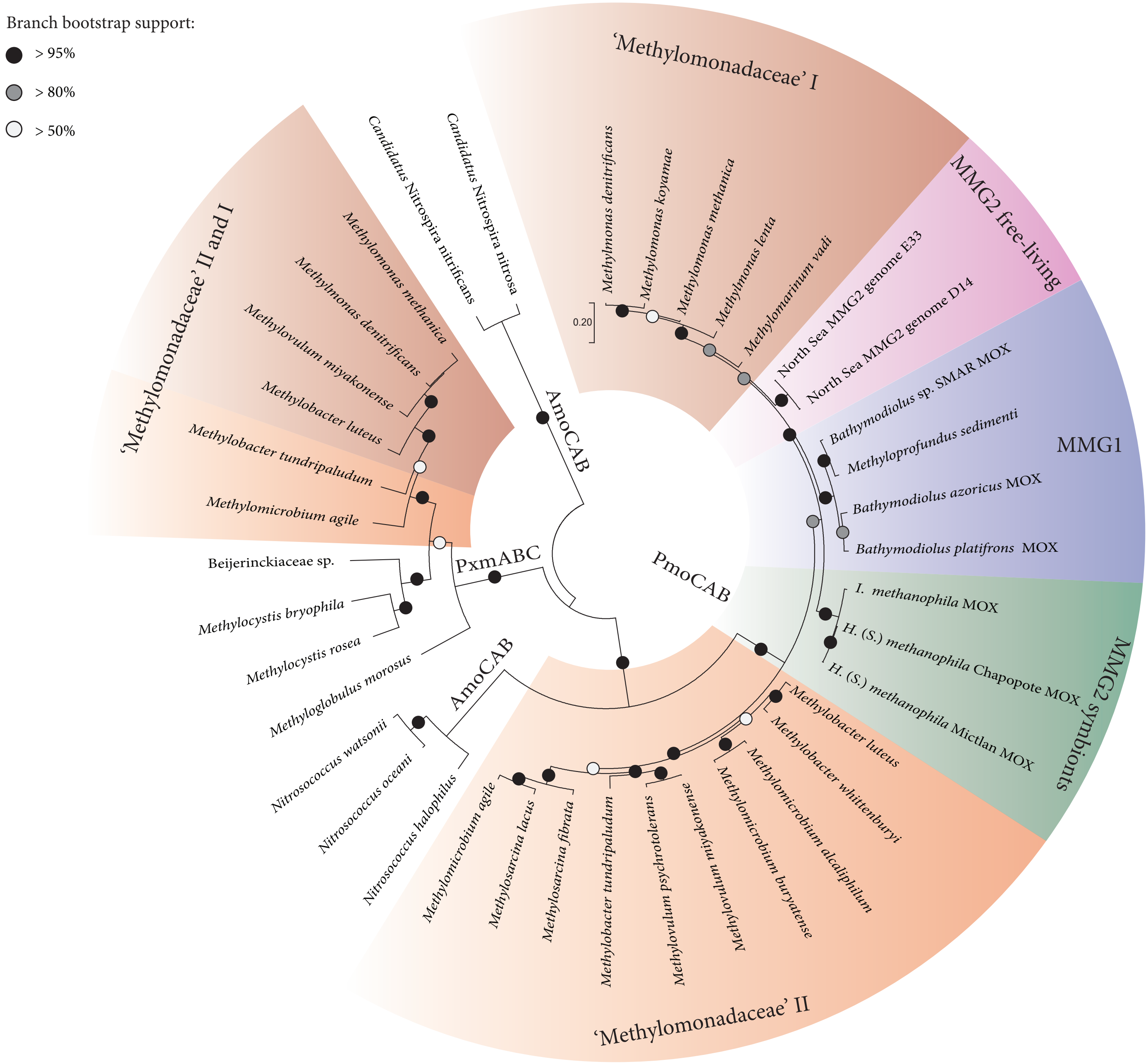

Supplementary Figure S4b Phylogeny of concatenated PmoCAB and PxmABC proteins (39 amino acid sequences total). Sequences were first aligned for each subunit, following concatenation of individual alignments in the ABC order. The tree is drawn to scale, with branch lengths measured in the number of substitutions per site. The analysis included 886 positions.

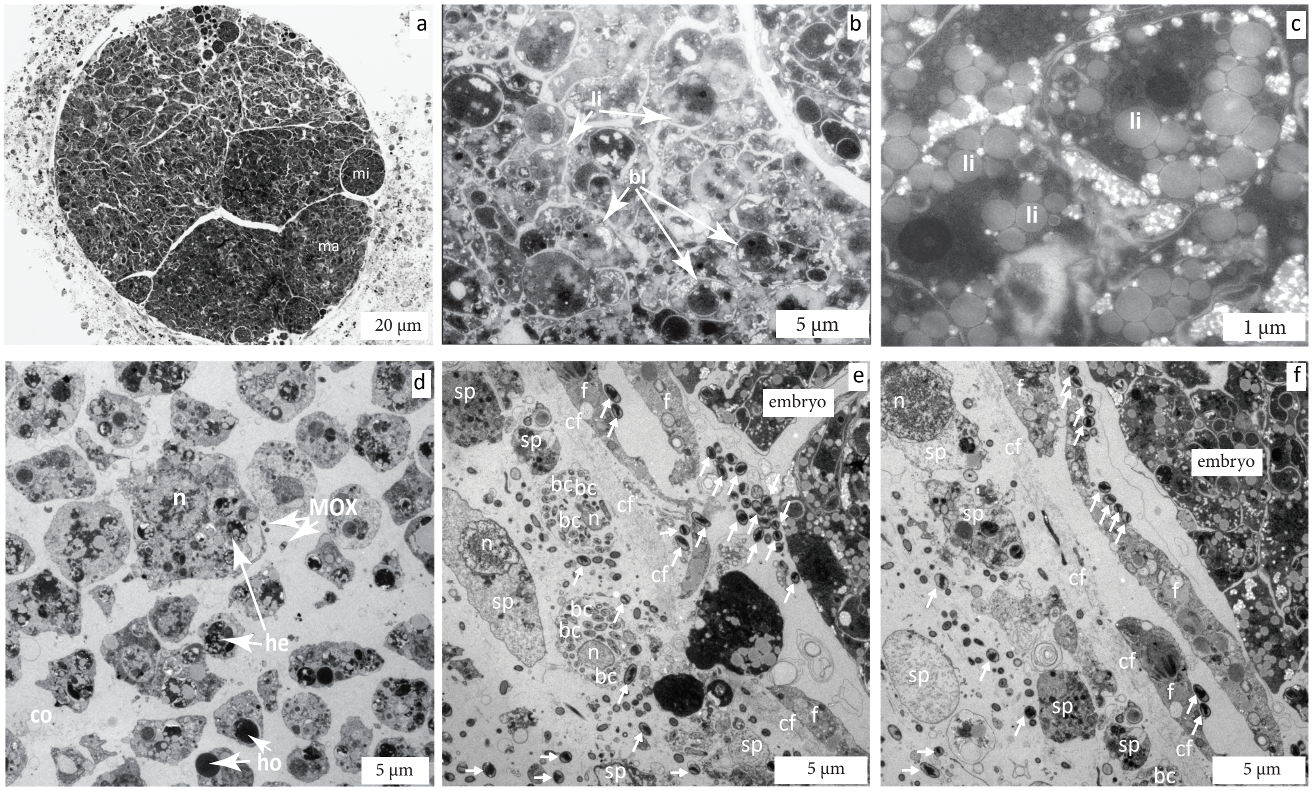

Supplementary Figure S5 TEM images of *H. (S.) methanophila* embryo tissue **a,** Overview of an embryo. *mi*=micromere cells, *ma*=macromere. **b,** electron-dense blastomeres (bl) with lipid droplet inclusions (*li*). **c,** Lipid droplet inclusions in blastomeres. **d,** nucleolated larval cells with homogeneous (*ho*) and heterogeneous yolk (*he*), *n* = nucleus, *co* = collagen fibers. **e and f,** methane-oxidizing symbionts accumulated between follicle-like cells (*f*) and embryo, and on the periphery of follicle-like cells. *bc*=bacteria in bacteriocytes; *cf*= collagen fibrils; *n*=nucleus; *sp*=sponge cell.

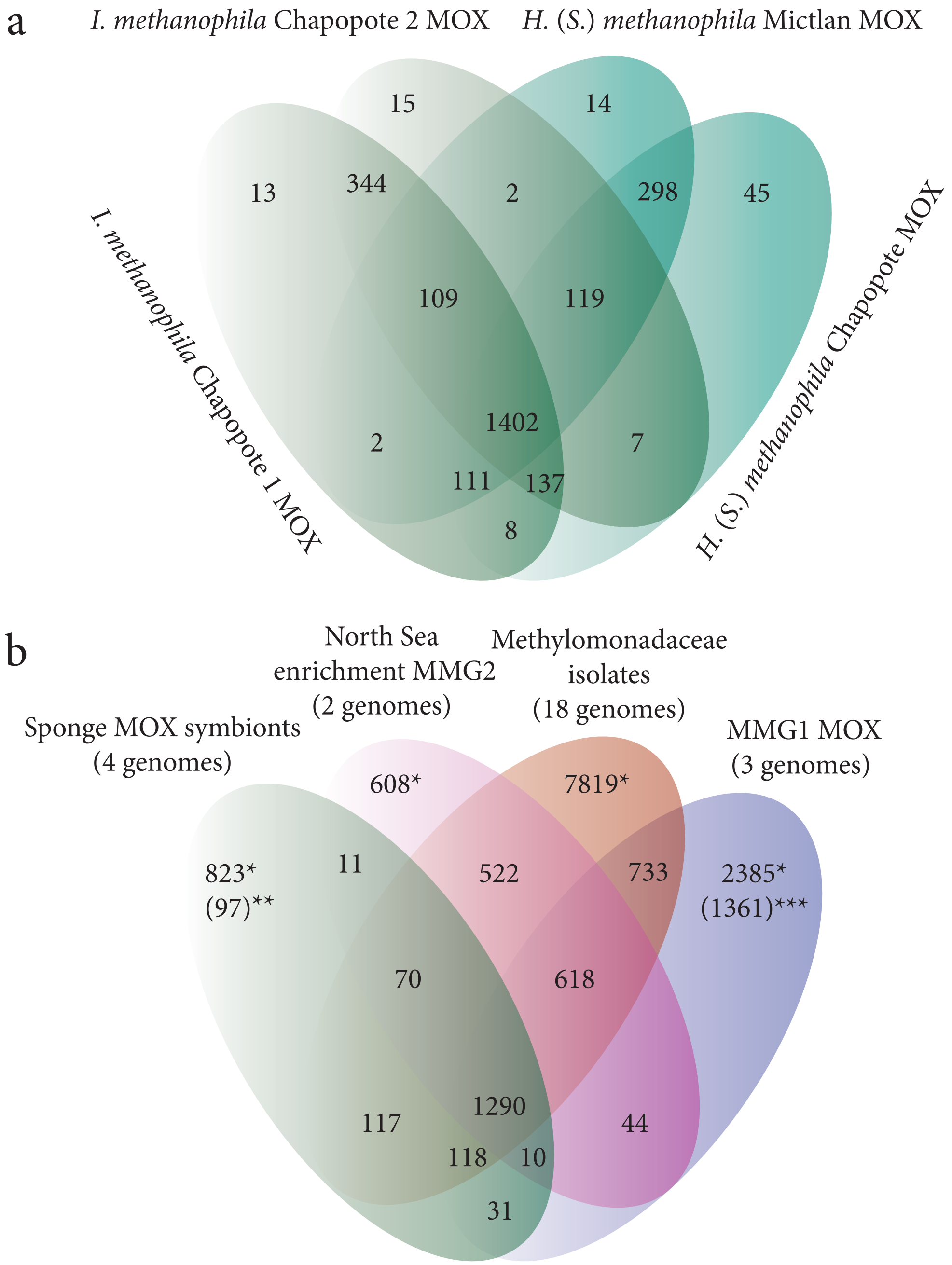

Supplementary Figure S6 Pangenome analysis of methane-oxidizing bacteria (MOX). Orthologous gene counts are shown. **a**, Comparison of the sponge MOX symbiont genomes assembled in this study. **b**, Comparison between the genomes of the sponge MOX symbionts and related free-living and symbiotic MOX. "Sponge MOX symbionts" group includes the genomes of MOX from the four sponge individuals analyzed in this study. "North Sea enrichment MMG2" group includes the two genomes of the Marine Methylotrophic Group 2 bacteria enriched from the North Sea sediments. "Methylomonadaceae isolates" group comprises the 18 representative genomes of Methylomonadaceae (main text, Figure 4), which were downloaded from NCBI. "MMG1" group comprises genomes of *Methyloprofundus sedimenti*, as well as those of MOX hosted by mussels *Bathymodiolus* sp. SMAR and *B. platifrons*. * clade-specific genes; ** unique to the sponge MOX symbiont core genome; *** mussel symbionts only (not including *M. sedimenti*).

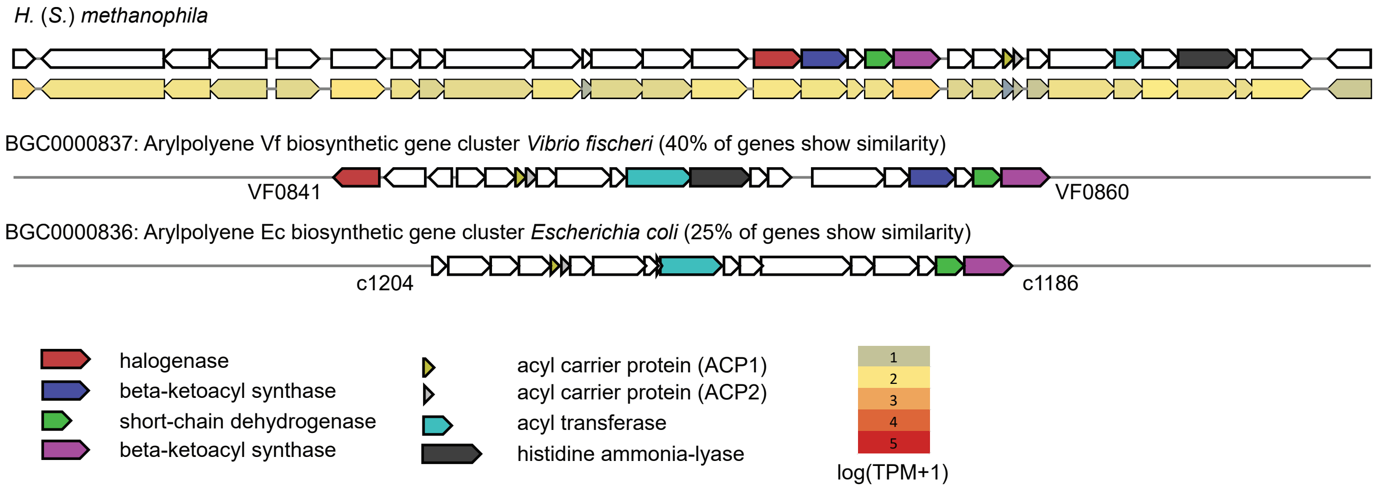

Supplementary Figure S7: Aryl polyene synthesis cluster in *H. (S.) methanophila* MOX symbiont genome. *Vibrio fischeri* and *Escherichia coli* Vf and Ec aryl polyene synthesis domains are shown as references. Homologs are shown in the same color. The second alignment is colored according to the average expression values of respective genes (color legend is shown in figure on bottom right).

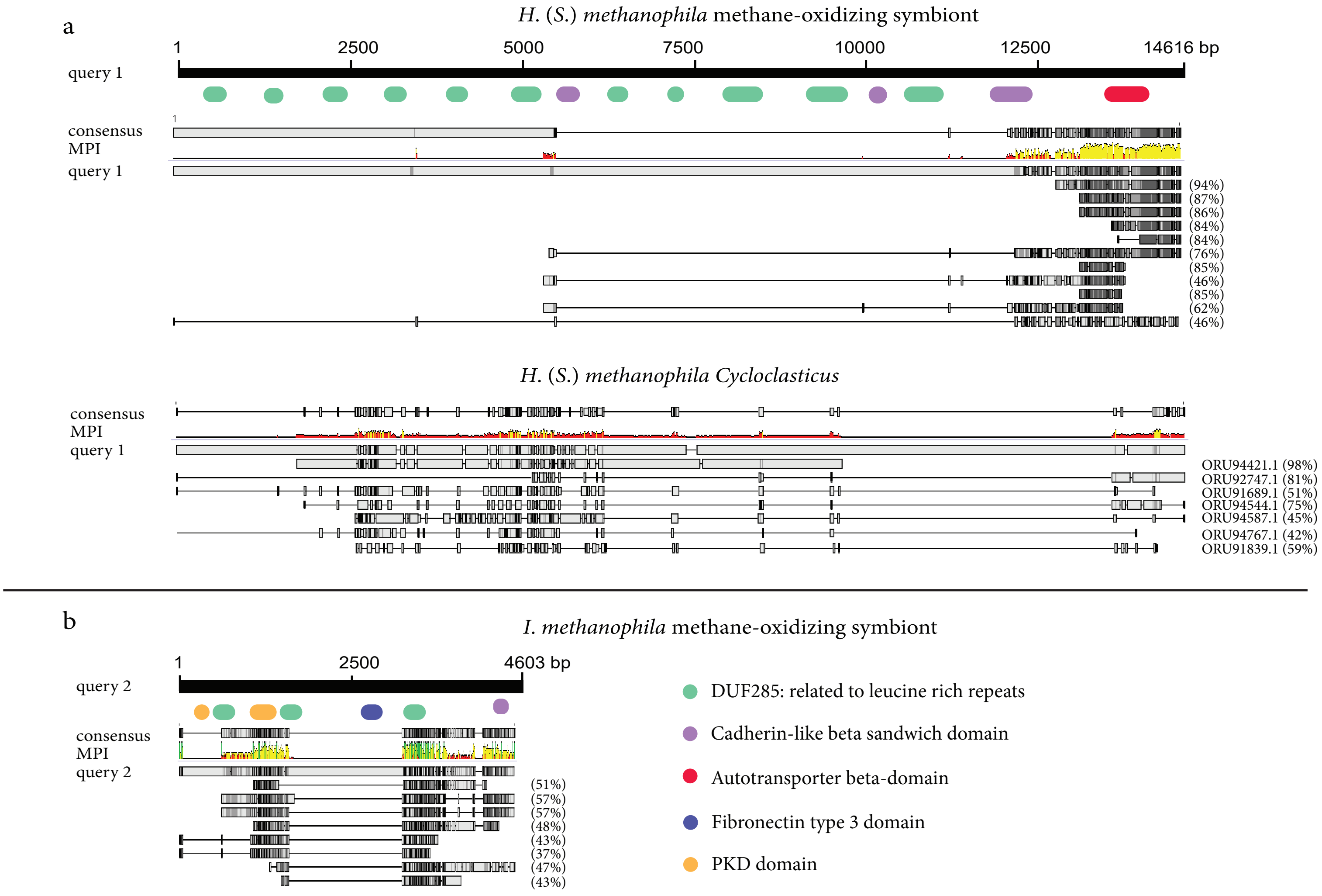

Supplementary Figure S8 (a,b) Structure of and occurrence of eukaryote-like protein (ELP)-encoding sequences in the genomes of sponge symbionts (a, b) and in sponge metagenomes (c, d). **a,** The structure of the longest ELP in *H.* (*S.*) *methanophila* MOX symbiont. Eukaryote-like domains (ELDs), which comprised leucine-rich repeats and cadherin-like domains, as well as an autotransporter beta-domain, which encodes a pore-forming element of the type V secretion system are shown. BLAST hits for this sequence (query 1) within the genome of *H.* (*S.*) *methanophila* MOX and *Cycloclasticus* symbionts are shown below. **b**, The structure of the longest ELP in *I. methanophila* MOX symbiont. Eukaryote-like domains (ELDs), which comprised leucine-rich repeats cadherin-like, polycystic kidney disease (PKD) and fibronectin type 3 domains are shown. BLAST hits for this sequence (query 2) within the genome of *H.* (*S.*) *methanophila* MOX are shown below. In panels **a** and **b**, identity values at amino acid level between the query and the sequences identified by BLAST are shown in parentheses. Consensus sequence and the mean pairwise identity (MPI) over all pairs in the column are included (100%, green; 30-99%, yellow; below 30%, red).

c

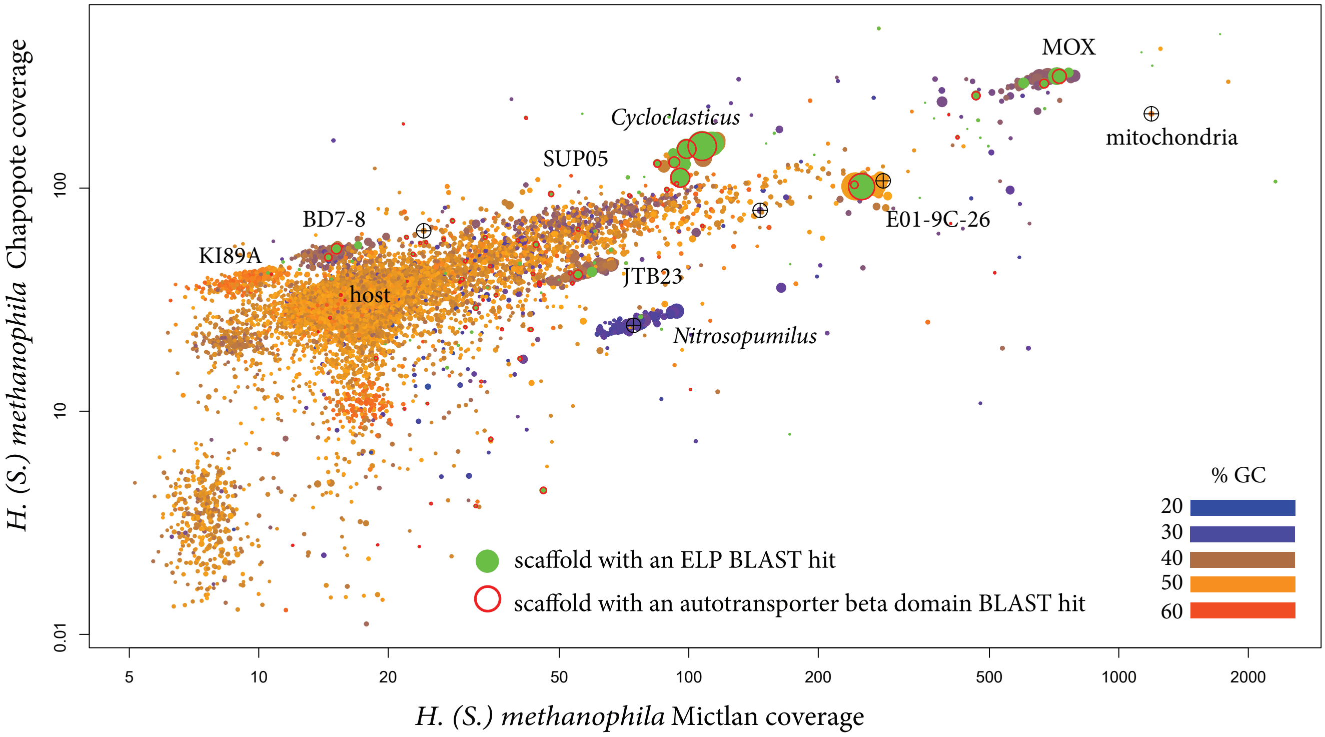

d

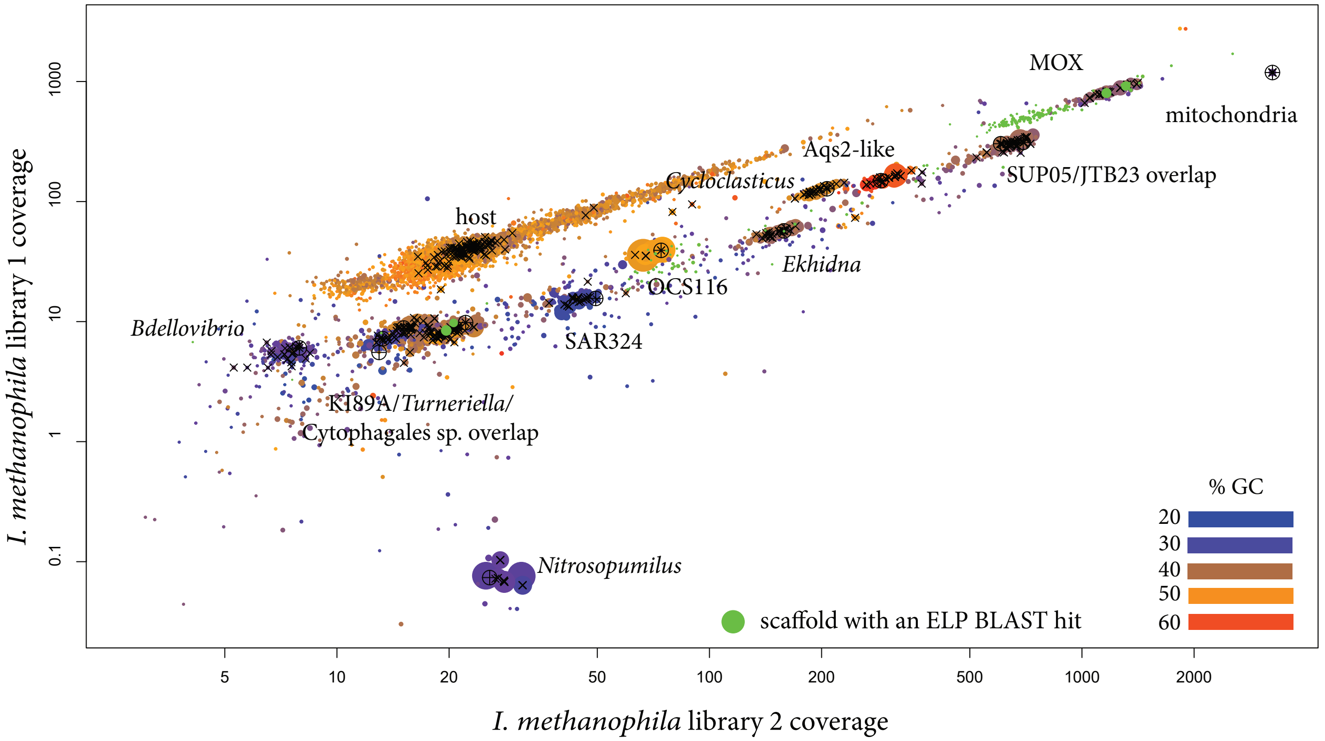

Supplementary Figure S8 (c, d) Structure of and occurrence of eukaryote-like protein (ELP)-encoding sequences in the genomes of sponge symbionts (a, b) and in sponge metagenomes (c, d). **c, d**: Scaffolds that contain ELPs (green circles) in the metagenomes of *H.* (*S.*) *methanophila* (c) and *I. methanophila* (d), revealed by BLAST analysis using the longest assembled ELP genes as queries. In panel c, red circles mark scaffolds that contain the autotransporter beta domains identified by BLAST using the respective partial ELP sequence as a query.

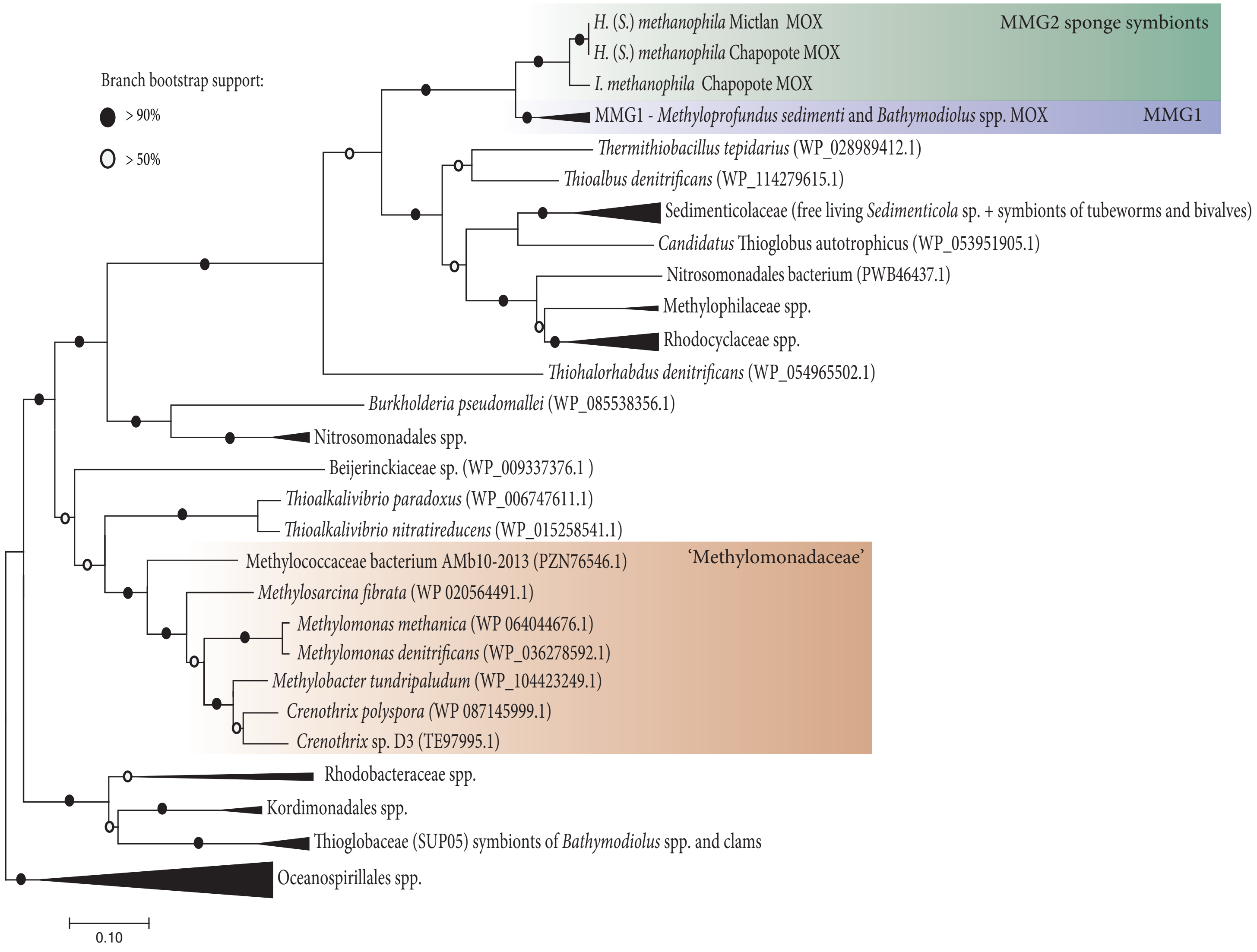

Supplementary Figure S9 Phylogeny of *narG* genes from the symbiotic MOX of sponges collected at the Campeche and Mictlan seeps (highlighted in green). The dataset included *narG* sequences from this study and from the NCBI database (73 amino sequences total). The tree is drawn to scale, with branch lengths representing the number of substitutions per site. The analysis included 1167 positions.

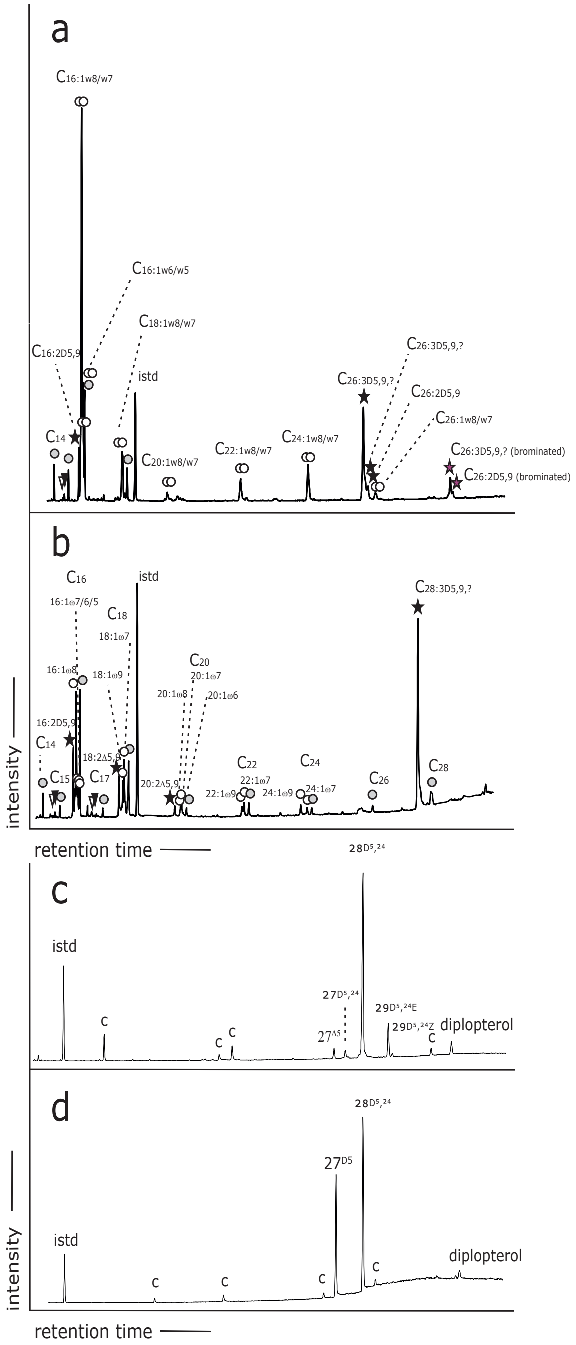

Supplementary Figure S10 Partial gas chromatograms (total ion current) of fatty acid (**a, b**) and alcohol fractions (**c, d**) in *H. (S.) methanophila* (**a, c**) and *I. methanophila* (**b, d**). Istd= internal standard. Symbols and detailed nomenclature; fatty acids (**a, b**): gray circles= saturated fatty acids, white circles= monounsaturated fatty acids, black stars= demospongic acids, red stars= brominated demospongic acids, white triangle= *iso*-fatty acids, black triangle= *anteiso*-fatty acids, ω= position of double bond counting from the methyl-end of the chain, Δ= position of double bonds counting from the carboxylic acid end. Nomenclature alcohols (c, d): $27^{\Delta5}$=cholest-5-*en*-3β-ol (cholesterol), $27^{\Delta5,24}$= cholesta-5,24-*dien*-3β-ol (desmosterol), $28^{\Delta5,24}$= 24-methylcholesta-5,24-*dien*-3β-ol, $29^{\Delta5,24}$= 24-ethylcholesta-5,24*E*-*dien*-3β-ol (fucosterol), $28^{\Delta5,24}$= 24-ethylcholesta-5,24*Z*-*dien*-3β-ol (*iso*-fucosterol), c= contaminants.

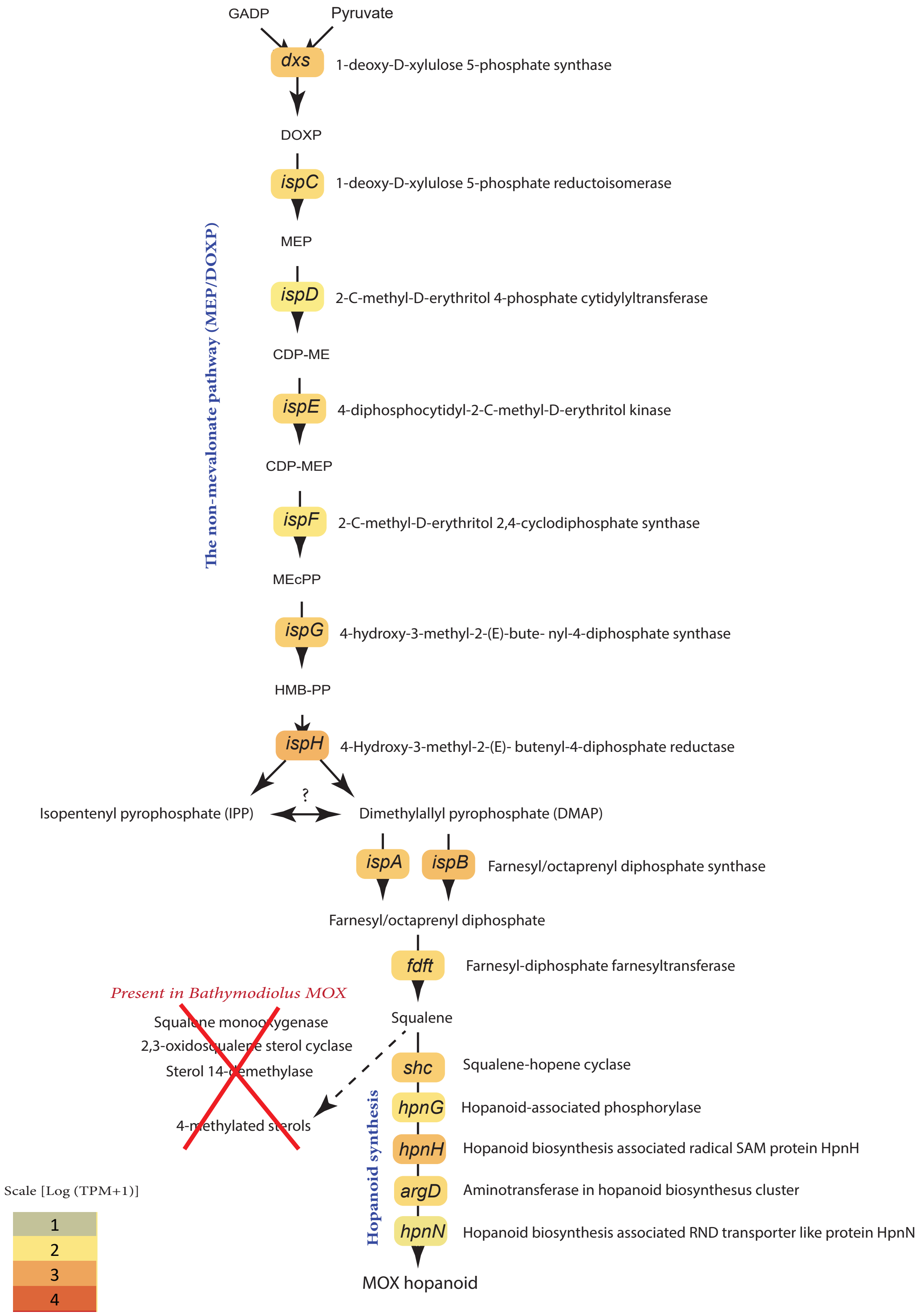

Supplementary Figure S11 Hopanoid synthesis in sponge MOX symbionts. The reconstruction is based on genomes of *I. methanophila* and *H.* (*S.*) *methanophila* MOX and on transcriptomes of *H.* (*S.*) *methanophila* MOX. Enzymes and their subunits are abbreviated with the name of the encoding gene. Boxes are colored according to the expression value of a respective transcript (color legend for expression values is shown on the bottom of figure).
